# An Effector TaCRVP From *Tuta Absoluta* Oral Secretion Impairs H_2_O_2_‐Mediated Plant Defense by Targeting Tomato SlCAT2

**DOI:** 10.1002/advs.77331

**Published:** 2026-08-25

**Authors:** Jianquan Yan, Youdan Zhang, Zhujun Wang, Huatao Tang, Huiping Liu, Zi Chen, Shuai Liu, Jiayi Shi, Lu Peng, Youming Hou

**Affiliations:** ^1^ State Key Laboratory of Agricultural and Forestry Biosecurity Fujian Agriculture and Forestry University Fuzhou Fujian China

**Keywords:** Catalase, Effector, Hydrogen peroxide, Oral secretions, Plant defense, Tuta absoluta

## Abstract

The arms race between plants and herbivorous insects involves sophisticated molecular strategies. While insects commonly secrete salivary effectors to modulate plant defenses, the underlying molecular mechanisms remain poorly characterized. In particular, as a devastating solanaceous pest, how *Tuta absoluta*, precisely subverts host immunity remains largely unknown. Here, we identify a novel salivary effector, the cysteine‐rich venom protein (TaCRVP), from the oral secretions (OSs) of *T. absoluta*. We reveal that TaCRVP is translocated into plant cells where it effectively hijacks host redox homeostasis by targeting the tomato catalase SlCAT2. Specifically, TaCRVP not only directly activates SlCAT2 H_2_O_2_‐scavenging capacity by promoting its tetramerization, but also blocks it from 26S proteasomal degradation by competitively inhibiting its interaction with the host E3 ubiquitin ligase SlAdBiL. Through this sophisticated dual mechanism, TaCRVP enhances SlCAT2 activity and stability to effectively suppress host H_2_O_2_ bursts, thereby subverting downstream jasmonic acid (JA) signaling and defensive metabolite production to facilitate insect feeding. Collectively, our results reveal a delicate evolutionary strategy whereby an insect effector hijacks the host SlCAT2‐SlAdBiL regulatory module, contributing to insect adaptation to host plants.

## Graphical abstract

The *Tuta absoluta* salivary effector TaCRVP subverts tomato immunity by hijacking host catalase SlCAT2. Through a sophisticated dual mechanism, TaCRVP simultaneously promotes SlCAT2 tetramerization for activation and blocks its SlAdBiL‐mediated degradation for stabilization. This synergistic regulation suppresses host H_2_O_2_ bursts and jasmonic acid signaling, ultimately facilitating successful insect feeding.

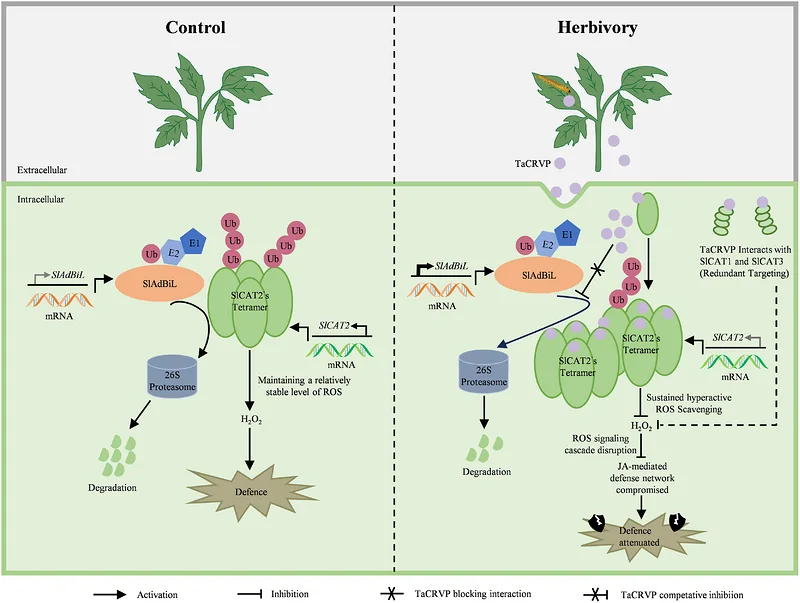

## 1 Introduction

The evolutionary arms race between herbivorous insects and host plants is shaped by the rapid activation of plant immune responses [1, 2]. A pivotal, early event in this defense network is the reactive oxygen species (ROS) burst [3, 4]. Beyond acting as a direct physical and chemical deterrent to feeding [5], ROS serve as crucial secondary messengers that mediate calcium fluxes, mitogen‐activated protein kinase (MAPK) cascades, and jasmonic acid (JA) signaling to restrict insect performance [6, 7, 8]. Consequently, the ability of herbivorous insects to rapidly suppress or detoxify the host ROS burst is a major determinant of their feeding success and survival.

To prevent self‐inflicted oxidative damage, plants maintain redox homeostasis through complex antioxidant systems, with catalase (CAT) acting as the primary enzymatic scavenger of hydrogen peroxide (H_2_O_2_) [8, 9]. While the exploitation of host CATs and ubiquitin‐proteasome systems by microbial effectors has been extensively characterized in plant‐pathogen interactions [10, 11, 12, 13], their specific regulatory roles during herbivory are poorly understood. However, whether similar molecular strategies apply to plant‐herbivore interactions remains a critical open question. While sap‐sucking insects (e.g., aphids) deliver effectors via stylets in a manner analogous to pathogens [14, 15, 16], tissue‐chewing lepidopteran pests cause catastrophic physical damage to host cells. It remains largely unknown whether such chewing herbivores possess the capacity to actively target host CATs to execute precise, intracellular manipulations of redox homeostasis and protein stability [17, 18, 19].

*Tuta absoluta* (Meyrick) is a devastating lepidopteran pest of solanaceous crops [20], yet the molecular mechanisms underlying its evasion of host immunity are poorly understood. In this study, we identify a novel cysteine‐rich venom protein (TaCRVP) from *T. absoluta* oral secretions and reveal its molecular mechanism in tomato. We demonstrate that TaCRVP employs a dual‐hijacking strategy directed at the tomato catalase SlCAT2 to subvert the host redox state. Upon translocation into plant cells, TaCRVP physically targets SlCAT2, not only activating its H_2_O_2_‐scavenging capacity by promoting essential tetramerization but also shielding it from 26S proteasomal degradation by competitively inhibiting the host E3 ubiquitin ligase SlAdBiL. By enhancing the activity and stability of SlCAT2, TaCRVP effectively suppresses the host ROS burst, thereby blocking downstream JA signaling and defense responses. These findings uncover a novel co‐evolutionary paradigm of an insect effector exploiting the host CAT enzyme via a multi‐layered regulatory strategy to subvert host redox immunity, thereby providing a critical theoretical for developing resistant crops against *T. absoluta*.

## 2 Results

### 2.1 TaCRVP Is Identified as a Secreted Salivary Effector From *T. absoluta*

Insect oral secretions (OSs) are key mediators of plant‐herbivore interactions, as they contain salivary effectors that modulate plant defenses. To gain deeper insight into the molecular basis of the *T. absoluta*‐tomato interaction, we performed liquid chromatography‐tandem mass spectrometry (LC‐MS/MS) to profile the OSs proteome (Figure S1A). While venom proteins are classically recognized as virulence factors injected by insect parasitoids to manipulate host immunity and development [21], recent evidence suggests they also act as critical effectors in herbivorous insects. A key example is HARP1, a novel effector in the OSs of *Helicoverpa armigera*, which shares homology with venom R‐like proteins from *Nasonia vitripennis* and functions to suppress plant defenses [17, 22]. Guided by these findings, we analyzed the *T. absoluta* OSs proteome and successfully identified two candidate venom proteins. Among them, an SCP domain‐containing protein, annotated as a cysteine‐rich venom protein and designated as TaCRVP, was prioritized for functional characterization because it exhibited a higher number of unique peptides and higher sequence coverage in the OSs proteome (Figure S1B).

To establish the in planta function of TaCRVP, we used an *Agrobacterium tumefaciens*‐mediated transient expression system in *Nicotiana benthamiana* leaves. Successful overexpression of TaCRVP was confirmed via Western blotting (Figure S2A). Subsequent bioassays revealed that larvae consumed a greater leaf area and showed enhanced weight gain on plants overexpressing TaCRVP relative to the controls (Figure S2B). To further elucidate its defense‐suppressing mechanism, we assessed its impact on pattern‐triggered immunity (PTI) [23]. Strikingly, compared to the GFP control, transient expression of TaCRVP‐GFP severely impaired the flg22‐induced ROS burst in tobacco leaves (Figure S2C, D). Collectively, these findings demonstrate that TaCRVP acts as a salivary effector that disrupts early plant defense signaling to promote *T. absoluta* performance and fitness.

**FIGURE 1:**
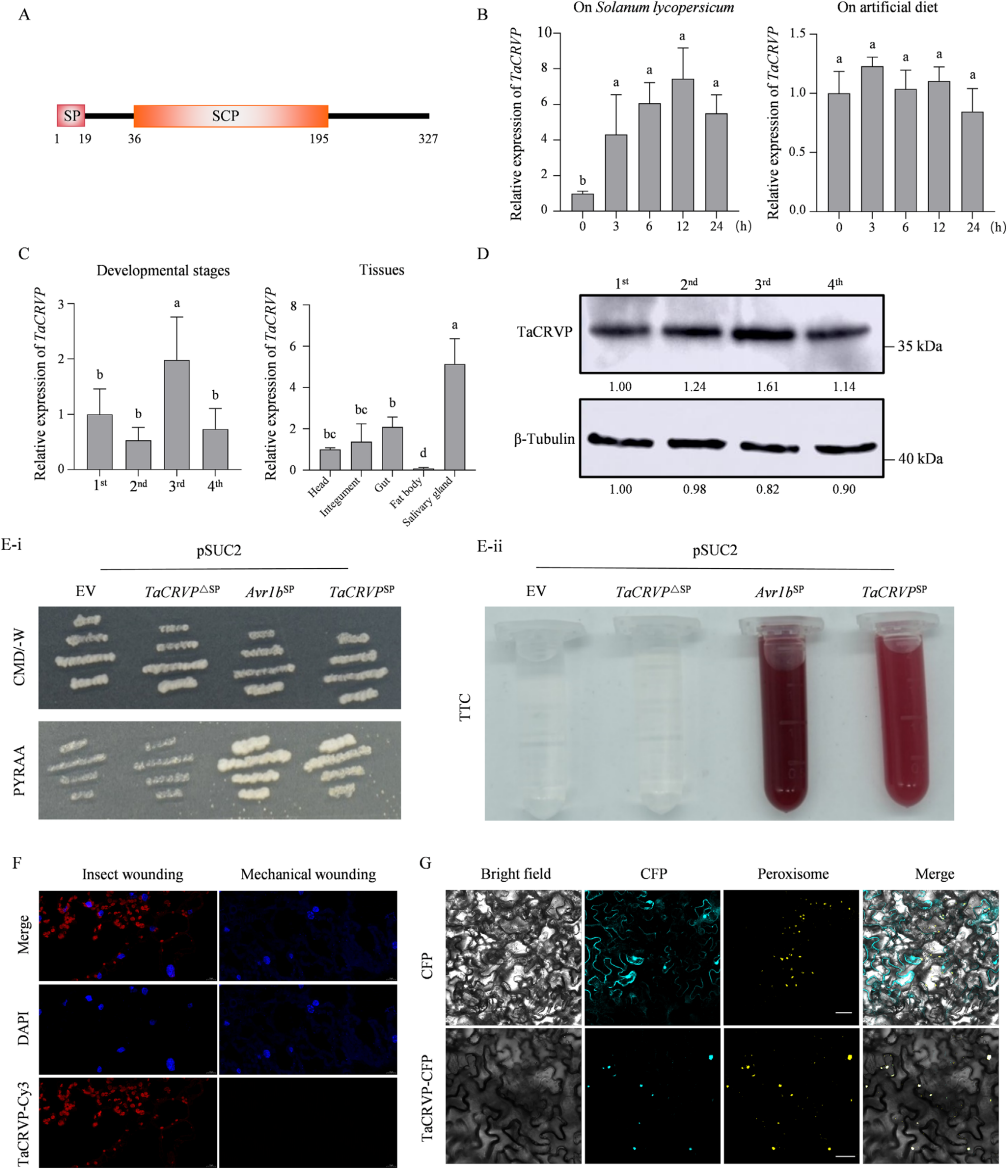
Identification of TaCRVP as a secreted salivary effector from *T. absoluta*. (A) Domain architecture of TaCRVP, featuring an N‐terminal signal peptide (residues 1–19) and an SCP domain (residues 36–195). (B) Time‐course transcript levels of *TaCRVP* in *T. absoluta* larvae feeding on an artificial diet vs. tomato plants (0, 3, 6, 12, and 24 h post‐feeding). Data are normalized to the 0‐h time point and the internal reference gene *EF1α*. (C) Tissue‐ and stage‐specific expression profiles of *TaCRVP*. Expression levels are normalized to head tissues and first‐instar larvae, respectively, using *SlActin* as the internal reference. (D) Immunoblot analysis of *T. absoluta* TaCRVP (36.4 kDa) in total protein extracts from whole larvae across different developmental stages. Exactly 30 µg of total crude protein was loaded per lane. β‐Tubulin serves as the loading control. The original blots can be found in Figure S1. (E) Functional validation of the TaCRVP SP using a yeast secretion trap assay. The predicted SP sequence (*TaCRVP* ^SP^) and the truncated mutant lacking the SP (*TaCRVP* ^ΔSP^) were cloned into the pSUC2 vector and transformed into the *Saccharomyces cerevisiae* strain YTK12. The empty vector (EV) and the *Avr1b* ^SP^ served as the negative and positive controls, respectively. Cell growth on the CMD/‐W medium confirms successful yeast transformation. Growth on the YPRAA medium (containing raffinose and antimycin A) and the conversion of TTC to a red precipitate indicate successful secretion of invertase, demonstrating the secretory function of the *TaCRVP* ^SP^. (F) Immunofluorescence localization of TaCRVP in host tissues. Representative images of whole‐mount Immunofluorescence assays on tomato leaves subjected to *T. absoluta* feeding (Insect wounding) or mechanical damage (Mechanical wounding). The specific accumulation of TaCRVP (red signal) is exclusively detected at the insect feeding sites. Nuclei are counterstained with DAPI (blue). Scale bars = 10 µm. (G) Subcellular co‐localization of TaCRVP with a peroxisome marker *in planta*. *Nicotiana benthamiana* leaves were agroinfiltrated to transiently co‐express TaCRVP‐CFP alongside the specific peroxisome marker PTS1‐YFP. Confocal microscopy images demonstrate that TaCRVP‐CFP forms distinct punctate structures that perfectly co‐localize with the peroxisome marker. The CFP empty vector served as the negative control, exhibiting a diffuse distribution throughout the cytoplasm and nucleus. Scale bars = 50 µm. Data are presented as mean ± SD (n = 3). Statistical significance was determined using one‐way ANOVA followed by Tukey's HSD test (B, C). Different lowercase letters indicate statistically significant differences (*p* < 0.05).

The TaCRVP protein comprises 327 amino acids and features a predicted N‐terminus signal peptide alongside an SCP domain, but lacks transmembrane domains (Figure 1A; Figure S3). LC‐MS/MS analysis identified two unique peptides, TASNLFTK (8 aa) and TASNLFTKPK (10 aa), that perfectly matched the TaCRVP sequence, thereby confirming its presence in the oral secretions (Figure S4). *TaCRVP* transcription was robustly upregulated upon tomato feeding by *T. absoluta* larvae, an induction completely absent when larvae were reared on an artificial diet (Figure 1B). Tissue‐ and stage‐specific profiling revealed that *TaCRVP* transcripts were predominantly expressed in the salivary glands and gut, concurrently exhibiting highly specific expression during the third‐instar stage (Figure 1C). To characterize the spatial and temporal expression patterns of TaCRVP, we generated a custom rabbit polyclonal antibody against TaCRVP. The antibody specifically recognized a single band matching the expected molecular weight of TaCRVP in both the in vitro expressed recombinant protein and the total protein extract of *T. absoluta* (Figure S5A). To further confirm this specificity in vivo, we used RNA interference (RNAi) to knock down *TaCRVP* transcription. Western blot analysis demonstrated that dsRNA‐mediated silencing of *TaCRVP* significantly reduced the TaCRVP protein signal in *T. absoluta* larvae at various time points compared to the control (Figure S5B). Consistent with its transcript levels, Western blot analysis of total protein from whole larvae confirmed that TaCRVP protein accumulation was most abundant in the third‐instar stage (Figure 1D). Furthermore, a yeast signal sequence trap assay confirmed that the predicted TaCRVP signal peptide (SP) efficiently mediates secretion, comparable to the Avr1b positive control (Figure 1E). To definitively establish whether TaCRVP is delivered into host tissues during herbivory, we first conducted whole‐mount immunofluorescence assays on infested tomato leaves. The results showed distinct, localized accumulation of TaCRVP exclusively at larval feeding sites (Figure 1F). To further validate these finding, we performed immunohistochemistry assays (Figure S6). Consistent with the IF data, TaCRVP signals were distinctly detected at the feeding sites of wild‐type and *dsGFP*‐treated larvae, but were drastically diminished at sites fed upon by *dsTaCRVP*‐treated larvae. Collectively, this dual validation unequivocally confirms that TaCRVP is specifically secreted and deposited into tomato tissues during *T. absoluta* feeding. To elucidate the subcellular localization of TaCRVP within plant cells, we transiently expressed TaCRVP fused to cyan fluorescent protein (TaCRVP‐CFP) alongside a peroxisome marker in *N. benthamiana* leaves. The results showed punctate structures of TaCRVP‐CFP that co‐localized with the peroxisome marker, whereas the CFP empty vector control distributed diffusely throughout the cytoplasm and nucleus (Figure 1G). Thus, upon entering the host, TaCRVP specifically targets peroxisomes.

### 2.2 The Salivary Effector TaCRVP Disrupts JA‐ and ROS‐Mediated Defenses in Tomato

To elucidate the in vivo biological function of TaCRVP, we used a dsRNA‐soaking RNAi approach. To validate the efficacy of this method, we first monitored the systemic uptake and silencing dynamics. Larvae treated with fluorescently labeled dsRNA exhibited strong and wide spread fluorescence throughout the entire body at 12 h following the 10‐min soaking treatment, with intense signals specifically accumulating in the dissected gut and salivary glands (Figure S7A). A subsequent time‐course RT‐qPCR analysis demonstrated that *TaCRVP* transcripts were significantly downregulated within 12 h, achieving peak silencing efficiency (60–65% reduction) between 24 and 72 h, before the silencing effect gradually waned by 120 h (Figure S7B). These results defined a strict 24–72 h period for subsequent bioassays.

**FIGURE 2:**
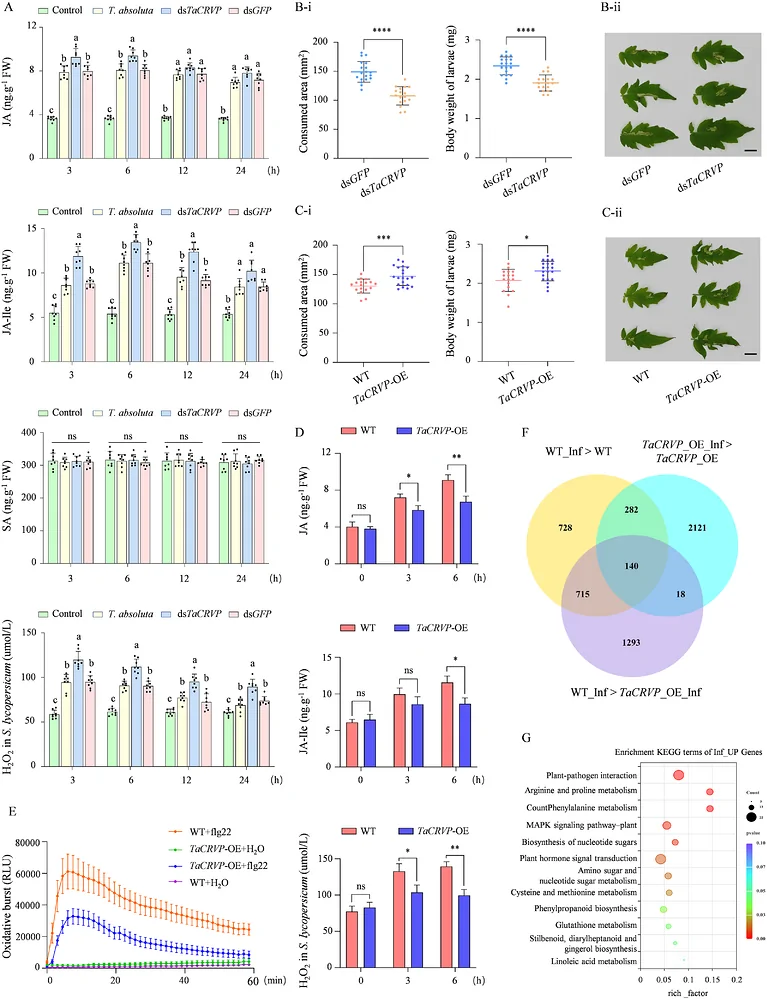
TaCRVP disrupts JA‐ and ROS‐mediated defenses in tomato. (A) Accumulation of defense signaling molecules (JA, JA‐Ile, H_2_O_2_, SA) in tomato plants subjected to different infestation treatments (uninfested control, wild‐type, ds*TaCRVP*‑treated *T. absoluta*, or ds*GFP*‑treated *T. absoluta*) at indicated time points post‐feeding (n = 8). (B) Bioassays of RNAi‐treated *T. absoluta* larvae. (B‐i) Leaf consumption area and larval body weight of ds*TaCRVP*‑ or ds*GFP*‑treated (control) third instars after feeding on tomato plants for 48 h (n = 17–20). (B‐ii) Representative images of the corresponding larvae after 48 h of feeding. Scale bars = 1 cm. (C) Bioassays of *T. absoluta* larvae on transgenic tomato plants. (C‐i) Leaf consumption area and larval body weight of *T. absoluta* after 48 h feeding on WT or *TaCRVP*‐OE tomato plants (n = 18–20). (C‐ii) Representative images of the corresponding larvae after 48 h feeding. Scale bars = 1 cm. (D) Accumulation of JA, JA‐Ile, and H_2_O_2_ in WT and *TaCRVP*‐OE tomato plants following *T. absoluta* infestation (n = 3). (E) Dynamic monitoring of flg22‐triggered ROS burst. Continuous ROS production was measured using a luminol‐based chemiluminescence assay in WT and *TaCRVP*‐OE leaf disks treated with flg22 or H_2_O. Data represent the mean ± SE (n = 12; RLU, relative light units). (F) Venn diagram illustrating the overlap of TaCRVP‐affected differentially expressed genes (DEGs) across the indicated comparative groups based on RNA‐seq analysis (n = 3). Group definitions are as follows: untreated WT tomato plants (WT), untreated *TaCRVP*‐OE plants (*TaCRVP*_OE), WT plants following 2 h of feeding by *T. absoluta* larvae (WT_Inf), and *TaCRVP*‐OE plants following 2 h of *T. absoluta* feeding (*TaCRVP*_OE_Inf). (G) KEGG pathway enrichment analysis of the specific DEGs within the Inf_UP group described in (F). The size of the circles represents the number of enriched genes, and the color gradient indicates the statistical significance (*p*‐value). Data are presented as mean ± SD. Multiple comparisons were analyzed using one‐way ANOVA followed by Tukey's HSD test (A). Different lowercase letters indicate statistically significant differences (*p* < 0.05). Statistical significance was determined using two‐tailed Student's *t*‐tests (B–D) (ns, not significant; *, *p* < 0.05; **, *p* < 0.01; ***, *p* < 0.001; ****, *p* < 0.0001).

Given that herbivore venom‐like proteins may function similarly to parasitoid venom proteins in targeting host defenses, we investigated whether TaCRVP suppresses tomato plant defense. To this end, we monitored the dynamics of key defense signaling molecules, including JA, jasmonoyl‐isoleucine (JA‐Ile), salicylic acid (SA), and H_2_O_2_. While wild‐type *T. absoluta* herbivory significantly induced JA, JA‐Ile, and H_2_O_2_ accumulation at multiple time points (3, 6, 12, and 24 h) without altering SA levels, infestation by ds*TaCRVP*‐treated larvae triggered an amplified induction of these defense signals compared to the ds*GFP* control (Figure 2A). We then evaluated the larval performance of the *TaCRVP*‐silenced larvae. Crucially, RNAi‐mediated knockdown of *TaCRVP* significantly restricted both leaf consumption and larval body weight gain on tomato plants (Figure 2B). However, no such weight difference was observed when larvae were reared on an artificial diet (Figure S8), unequivocally demonstrating that TaCRVP functions specifically as a crucial effector for overcoming plant defenses rather than a developmental regulator.

These loss‐of‐function results suggest that TaCRVP promotes tomato susceptibility by suppressing JA‐ and H_2_O_2_‐mediated defenses. To definitively establish whether the presence of TaCRVP alone is sufficient for this immunosuppression, we generated stable transgenic tomato lines constitutively overexpressing *TaCRVP* (*TaCRVP‐OE*). Immunoblot analysis confirmed robust protein accumulation (Figure S9A). Notably, while *TaCRVP*‐OE plants displayed normal developmental phenotypes comparable to wild‐type (WT) (Figure S9B, C), they were significantly more susceptible to *T. absoluta* (Figure 2C). Overexpression of TaCRVP severely impaired the herbivore‐induced bursts of JA, JA‐Ile, and H_2_O_2_ (Figure 2D, E). Collectively, these results unequivocally demonstrate that TaCRVP functions as a potent salivary effector protein to actively disrupt plant defense signaling.

To elucidate the downstream defense networks modulated by TaCRVP, we performed comparative RNA‐seq analysis on WT and *TaCRVP‐OE* tomato plants. Principal component analysis (PCA) and hierarchical clustering heatmaps revealed distinct expression patterns between the two genotypes in response to herbivory (Figure S10), clearly demonstrating that TaCRVP broadly affects host gene expression during insect feeding. Specifically, 2,166 genes exhibited significantly lower expression in infested *TaCRVP‐OE* plants relative to infested WT controls. Among these suppressed transcripts, 873 belonged to the herbivory‐induced gene set (Inf_UP), while the remaining 1,293 were unresponsive to feeding (Inf_Neutral) (Figure 2F; Table S2). KEGG pathway enrichment revealed that these downregulated genes are predominantly involved in core defense signaling, transcriptional regulation, physical barrier reinforcement, and secondary metabolite biosynthesis (Figure 2G). Furthermore, the RNA‐seq data indicated that the induction of multiple defense genes by *T. absoluta* was attenuated in *TaCRVP*‐OE plants (Figure S11A). These suppressed genes included those associated with physical defense (*SlKCS11*), JA signaling (*SlMYC2*, *SlLOX*, *SlJAZ3*), secondary metabolite synthesis (*SlPAL2*, *SlPHT*), and the protease inhibitor *SlPin1*. Finally, subsequent RT‐qPCR analysis fully corroborated the RNA‐seq results, confirming the significant attenuation of these herbivore‐induced defense genes upon *TaCRVP* overexpression (Figure S11B).

To investigate the impact of TaCRVP on tomato secondary metabolism, we performed untargeted metabolomic profiling of WT and *TaCRVP*‐OE plants in the presence or absence of *T. absoluta* herbivory. PCA revealed a clear separation between the metabolic profiles of WT and *TaCRVP*‐OE plants, confirming that TaCRVP expression reprograms the host metabolic response to herbivory (Figure S12A). Comparative analysis identified 122 secondary metabolites that were significantly less abundant in infested *TaCRVP*‐OE plants compared to infested WT plants. Specifically, 45 of these were herbivory‐induced, whereas 77 were feeding‐independent (Figure S12B and Table S3). Strikingly, a vast majority of these suppressed compounds serve as critical components of direct or indirect plant immunity, including key sesquiterpenes, diterpenes, sterol glycosides, flavonoids, and diverse fatty acid derivatives, alongside essential signaling precursors like linoleic acid and eicosanoids (Figure S12C). Consistent with our transcriptomic data, KEGG enrichment analysis demonstrated that these reduced metabolites are closely associated with core defense pathways, most notably linoleic and α‐linolenic acid metabolism, plant hormone signal transduction, and arachidonic acid metabolism (Figure S12D).

Integrated transcriptomic and metabolomic analyses systematically demonstrated that *T. absoluta* infestation triggers a robust JA‐mediated defense program in tomato. This multifaceted response comprises direct defenses (e.g., protease inhibitors), indirect defenses (e.g., fatty acid‐derived volatiles), and physical barrier reinforcement (e.g., cell wall thickening). Crucially, the salivary effector TaCRVP disrupts this defense network, causing a global suppression of host immunity that ultimately facilitates larval performance.

### 2.3 TaCRVP Targets the Host Catalase SlCAT2 to Suppress Plant Immunity

**FIGURE 3:**
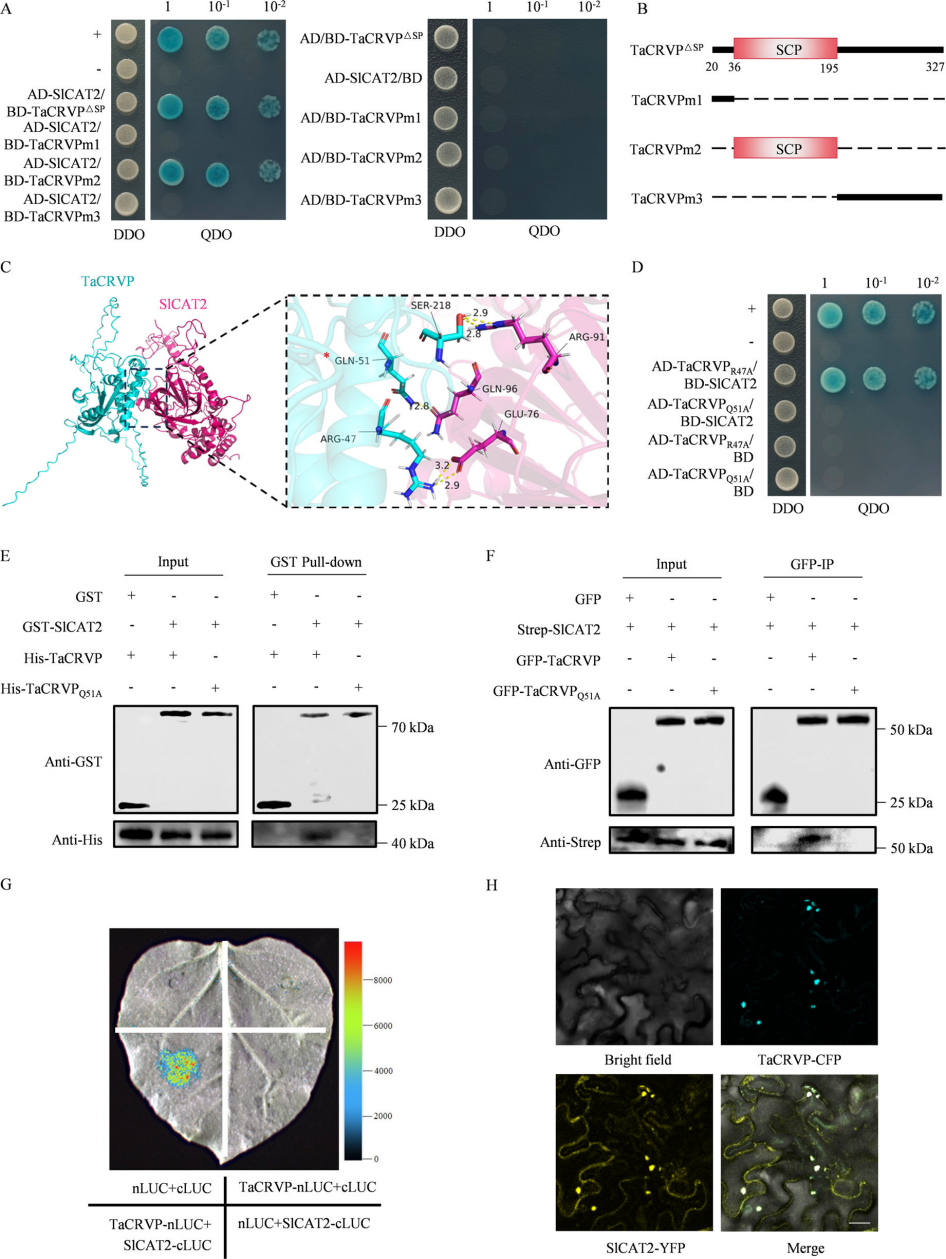
TaCRVP targets and physically interacts with the tomato catalase SlCAT2. (A) Y2H assays verifying the interaction between TaCRVP and SlCAT2. (B) Schematic representation of TaCRVP protein and its truncated mutants. (C) AlphaFold 3‐predicted structural model of the TaCRVP‐SlCAT2 complex. TaCRVP is shown in cyan, and SlCAT2 is shown in pink. The boxed area represents the magnified inset on the right. Residues R47, Q51, and S218 in TaCRVP were identified as potential key sites responsible for the interaction with SlCAT2. (D) Y2H assays verifying the interactions between TaCRVP_R47A_ or TaCRVP_Q51A_ and SlCAT2. For (A) and (D), yeast cells co‐transformed with the indicated bait and prey constructs were spotted onto selective media. Cell growth on the double dropout medium (DDO, SD/–Trp/–Leu) confirms successful co‐transformation, whereas growth and blue coloration on the quadruple dropout medium (QDO, SD/–Trp/–Leu/–His/–Ade) supplemented with X‐α‐Gal indicates physical protein‐protein interactions. Serially diluted yeast cultures (1, 10^−^^1^, and 10^−^^2^) are shown. Co‐transformants carrying corresponding empty AD or BD vectors served as auto‐activation controls. The combinations of pGBKT7‐53/pGADT7‐T and pGBKT7‐Lam/pGADT7‐T served as the positive (+) and negative (–) controls, respectively. (E) GST pull‐down assay validating the interaction between TaCRVP or TaCRVP_Q51A_ and SlCAT2. Recombinant SlCAT2‐GST, TaCRVP‐His and TaCRVP_Q51A_‐His proteins were expressed in and purified from *Escherichia coli* strain BL21. Total protein extracts (Input) and pull‐down fractions were analyzed by immunoblotting using anti‐GST and anti‐His antibodies. (F) Co‐IP assay validating the in planta interaction between TaCRVP or TaCRVP_Q51A_ and SlCAT2. *N. benthamiana* leaves were agroinfiltrated to transiently co‐express TaCRVP_Q51A_‐GFP or TaCRVP‐GFP alongside SlCAT2‐Strep. Input was subjected to immunoprecipitation (IP) using anti‐GFP agarose beads. Both the Input and IP fractions were analyzed by immunoblotting using anti‐GFP and anti‐Strep antibodies. (G) LCI assay validating the interaction between TaCRVP and SlCAT2. *N. benthamiana* leaves were co‐infiltrated with *Agrobacterium* suspensions harboring the TaCRVP‐nLuc and SlCAT2‐cLuc fusion constructs. Luminescence signals were captured at 48 h post‐infiltration after the application of D‐luciferin. Co‐expression of the target proteins with corresponding empty vectors served as negative controls. (H) Subcellular co‐localization of TaCRVP and SlCAT2 in planta. Confocal microscopy reveals co‐localization of TaCRVP‐CFP and SlCAT2‐YFP in *N. benthamiana* epidermal cells, as indicated by the white signal in the merged image. Scale bars = 20 µm. The original images for all immunoblots shown in this figure are provided in Figure S2.

To elucidate the molecular mechanisms underlying TaCRVP‐mediated immunosuppression, we performed a yeast two‐hybrid (Y2H) screen using a tomato cDNA library and identified the catalase SlCAT2 as a direct interactor (Figure 3A; Figure S13). Y2H assays using truncated TaCRVP variants (TaCRVPm1‐m3) revealed that the SCP domain‐containing fragment, TaCRVPm2, is both necessary and sufficient for this interaction (Figure 3A, B). Consistently, TaCRVPm2 retained the ability to suppress flg22‐induced PTI, confirming that the SCP domain mediates both target binding and defense suppression (Figure S14A, B). To uncover the structural basis of this complex, molecular docking was performed, predicting TaCRVP residues R47, Q51, and S218 to be situated at the binding interface (Figure 3C). Subsequent mutational analyses in yeast robustly validated this model, revealing that whereas an R47A substitution had no effect, a single Q51A mutation completely abrogated the interaction, identifying Q51 as the indispensable binding determinant (Figure 3D). This Q51‐dependent direct physical association was comprehensively corroborated in vitro and in vivo through glutathione S‐transferase (GST) pull‐down, Co‐immunoprecipitation (Co‐IP), luciferase complementation imaging (LCI), and co‐localization assays (Figure 3E–H). Crucially, the interaction‐deficient TaCRVP_Q51A_ mutant entirely lost its capacity to suppress flg22‐triggered immunity (Figure S14C, D), intrinsically linking SlCAT2 binding to effector function. Finally, investigating whether this interaction is conserved across the broader catalase family revealed that TaCRVP can also physically interact with SlCAT1 and SlCAT3, albeit with markedly weaker interaction intensities compared to SlCAT2 (Figure S15). This suggests an evolutionary strategy whereby TaCRVP broadly targets the conserved tomato catalase family, with SlCAT2 acting as its primary interacting node.

Because our initial bioassays demonstrating TaCRVP‐mediated feeding promotion and ROS suppression were conducted in *N. benthamiana*, we hypothesized that this effector might target homologous catalases in this experimental host to subvert immunity. Phylogenetic analysis identified the *N. benthamiana* catalase 01049 as the closest ortholog to *SlCAT2* (Figure S16A, B). Importantly, Y2H assays confirmed a direct physical interaction between TaCRVP and Nb_01049 (Figure S16C). Together, these results demonstrate that TaCRVP suppresses plant defenses via a conserved catalase‐targeting mechanism across different solanaceous hosts.

### 2.4 *SlCAT2* Negatively Regulates Tomato Resistance against *T. absoluta*

**FIGURE 4:**
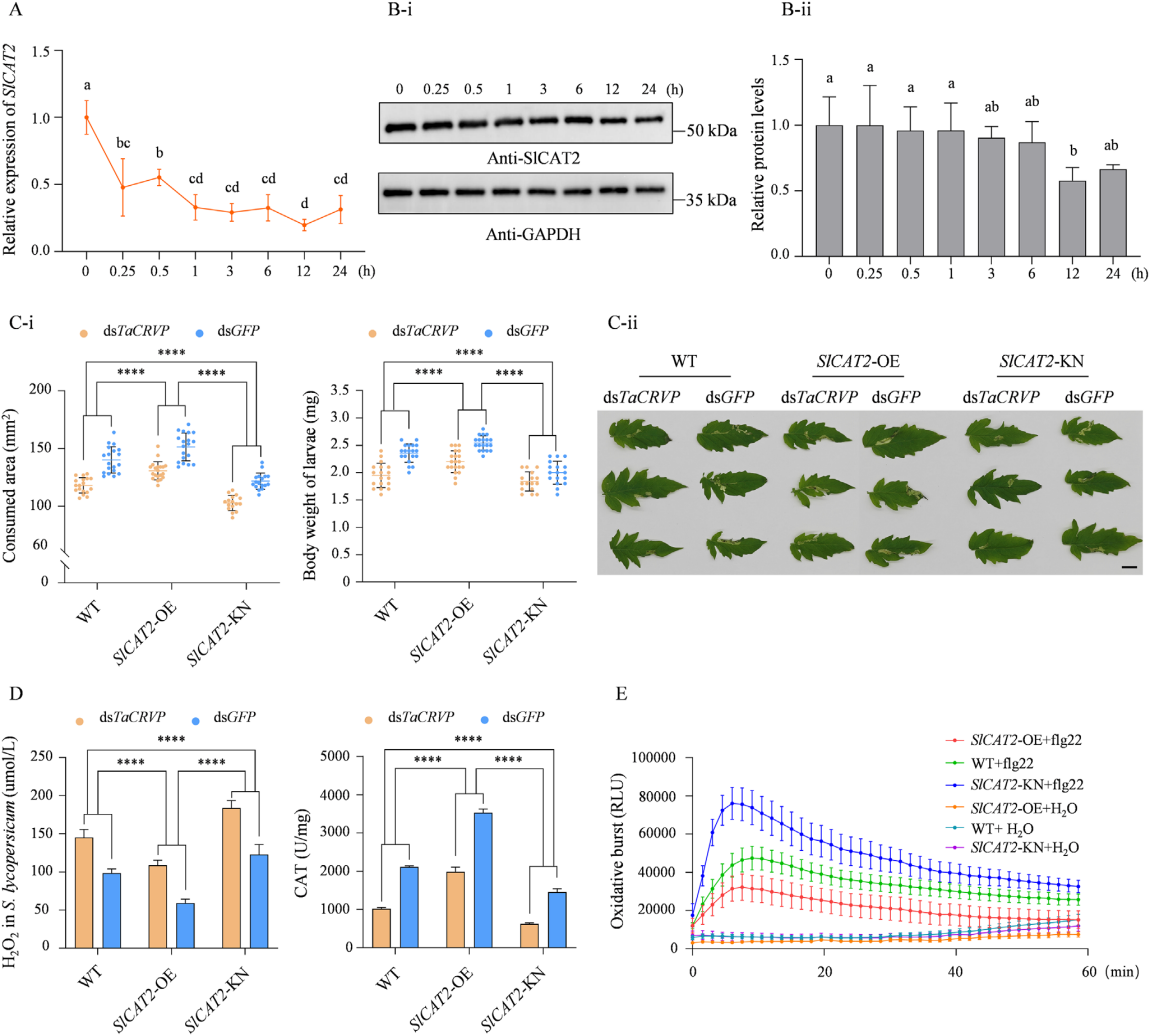
*SlCAT2* negatively regulates tomato resistance against *T. absoluta*. (A) Time‐course transcript levels of *SlCAT2* in tomato leaves following *T. absoluta* feeding (0, 0.25, 0.5, 1, 3, 6, 12, and 24 h post‐feeding) Data are normalized to the 0‐h time point and the internal reference gene *SlActin* (n = 3). (B) Time‐course accumulation of SlCAT2 protein in tomato leaves following *T. absoluta* feeding. (B‐i) Immunoblot analysis of SlCAT2 protein levels at 0, 0.25, 0.5, 1, 3, 6, 12, and 24 h post‐feeding. GAPDH served as the loading control. (B‐ii) Relative quantification of SlCAT2 protein levels normalized to GAPDH (n = 3). The original blots can be found in Figure S3. (C) Bioassays evaluating the performance of RNAi‐treated *T. absoluta* larvae on different tomato genotypes. (C‐i) Leaf consumption area and larval body weight of ds*TaCRVP* or ds*GFP*‑treated *T. absoluta* larvae after feeding on the WT, *SlCAT2*‐OE and *SlCAT2*‐KN tomato plants for 48 h (n = 16–20). (C‐ii) Representative images of the corresponding larvae described in (C‐i) following 48 h feeding on WT, *SlCAT2*‐OE, and *SlCAT2*‐KN tomato plants. Scale bars = 1 cm. (D) H_2_O_2_ accumulation and CAT enzyme activity in WT, *SlCAT2*‐OE, and *SlCAT2*‐KN tomato plants following 3 h of feeding by ds*TaCRVP*‐ or ds*GFP*‐treated *T. absoluta* larvae (n = 3). (F) Dynamic monitoring of flg22‐triggered ROS burst. Continuous ROS production was measured with a luminol‐based chemiluminescence assay in WT, *SlCAT2*‐OE, and *SlCAT2*‐KN leaf disks treated with flg22 or H_2_O. Data represent the mean ± SE (n = 12; RLU, relative light units). Data are presented as mean ± SD unless otherwise noted. Multiple comparisons were analyzed using one‐way ANOVA followed by Tukey's HSD test (A, B). Different lowercase letters indicate statistically significant differences (*p* < 0.05). Statistical significance was determined using two‐way ANOVA followed by Tukey's HSD test (C, D) (ns, not significant; *, *p* < 0.05; **, *p* < 0.01; ***, *p* < 0.001; ****, *p* < 0.0001).

To elucidate the temporal dynamics of *SlCAT2* during tomato defense, we quantified its transcript and protein levels upon *T. absoluta* herbivory. RT‐qPCR profiling revealed a rapid suppression of *SlCAT2* transcripts beginning at 0.25 h post‐infestation and persisting through 24 h (Figure 4A). In striking contrast, corresponding immunoblot analysis indicated that SlCAT2 protein levels remained remarkably stable during early feeding (0–6 h), experiencing only a modest reduction at later time points (12 and 24 h) (Figure 4B). The stark discrepancy between the severe transcriptional shutdown and the delayed decline in protein levels suggests a complex dual‐regulation mechanism. We hypothesize that this moderate decline in SlCAT2 protein is a balanced outcome between decreased transcription and an increase in protein stability during the plant‐insect interaction.

To validate the functional role of *SlCAT2* in this process, we generated overexpression (OE) and CRISPR/Cas9‐mediated knockout (KN) lines of *SlCAT2* (Figure S17A, B). While *SlCAT2*‐OE plants developed normally, the *SlCAT2*‐KN mutants displayed noticeably stunted growth (Figure S17C, D). In insect bioassays, *T. absoluta* larvae consumed more leaf area and gained more weight on *SlCAT2*‐OE plants, whereas both measures were significantly reduced on *SlCAT2*‐KN lines compared to the WT (Figure 4C). Furthermore, to investigate whether the effector function of TaCRVP depends on host SlCAT2 levels, we compared the fold changes in insect performance (ds*TaCRVP* vs. ds*GFP* treatments) across different plant genotypes. While the relative reduction in leaf area consumed was consistent across all lines (Figure S18A), the relative reduction in larval weight caused by *TaCRVP* silencing was significantly attenuated on *SlCAT2*‐KN plants compared to the WT and *SlCAT2*‐OE controls (Figure S18B). This differential response indicates that the effect of TaCRVP is functionally linked to the presence of SlCAT2. However, the observation that *TaCRVP* silencing still exerted a partial negative effect on larvae feeding on *SlCAT2*‐KN plants suggests that SlCAT2 is not its exclusive target. Consistent with these bioassays, the *SlCAT2*‐OE plants displayed decreased H_2_O_2_ accumulation and enhanced CAT activity relative to WT controls, whereas the *SlCAT2*‐KN lines exhibited the opposite phenotypes, confirming the central role of SlCAT2 in H_2_O_2_ scavenging (Figure 4D, E). Notably, silencing *TaCRVP* still led to a significant increase in H_2_O_2_ accumulation even in the *SlCAT2*‐KN background. We attribute this residual ROS suppression to target redundancy, whereby TaCRVP maintains partial ROS‐scavenging capacity through its physical interactions with SlCAT1 and SlCAT3 even in the absence of SlCAT2. Collectively, our bioassays and biochemical analyses of different *SlCAT2* genotypes demonstrate that this catalase is the principal target manipulated by the herbivore to compromise host resistance, while target redundancy within the family ensures resilient ROS suppression.

### 2.5 SlAdBiL Promotes Tomato Immunity by Targeting SlCAT2 for Ubiquitination and Degradation

**FIGURE 5:**
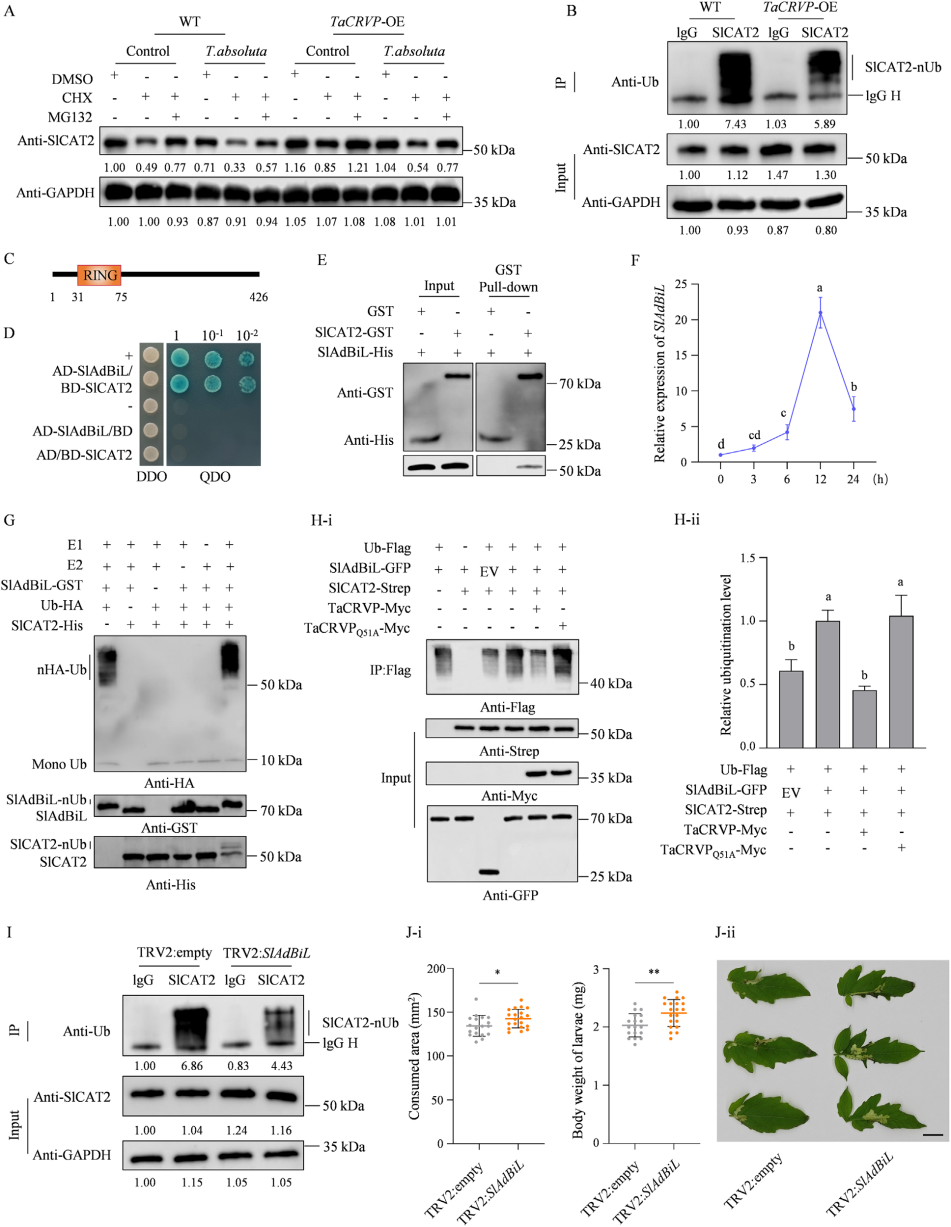
SlAdBiL promotes tomato immunity by targeting SlCAT2 for ubiquitination and degradation. (A) Degradation dynamics of SlCAT2 proteins. WT and *TaCRVP*‐OE tomato leaves were infiltrated with DMSO, 50 µM MG132, or 75 µM CHX for 6 h, followed by 12 h of feeding with or without *T. absoluta*. Total proteins were isolated for immunoblot analysis. GAPDH served as the loading control. (B) Effect of TaCRVP on the in vivo ubiquitination of SlCAT2. Total proteins from WT and *TaCRVP*‐OE plants were immunoprecipitated with an anti‐SlCAT2 antibody or control IgG, followed by immunoblotting with an anti‐Ub antibody. Input were subjected to immunoblot analysis using anti‐SlCAT2 and anti‐GAPDH antibodies. (C) Schematic representation of the domain architecture of the E3 ubiquitin ligase SlAdBiL. (D) Y2H assays verifying the interaction between SlCAT2 and SlAdBiL. Yeast cells co‐transformed with the bait construct (BD‐SlCAT2) and prey construct (AD‐SlAdBiL) were spotted onto selective media. Cell growth on the double dropout medium (DDO, SD/–Trp/–Leu) confirms successful co‐transformation. The growth and blue coloration of yeast colonies on the quadruple dropout medium (QDO, SD/–Trp/–Leu/–His/–Ade) supplemented with X‐α‐Gal indicates a physical protein‐protein interaction. Serially diluted yeast cultures (1, 10^−^^1^, and 10^−^^2^) are shown. Co‐transformants carrying corresponding empty AD or BD vectors served as auto‐activation controls. The combinations of pGBKT7‐53/pGADT7‐T and pGBKT7‐Lam/pGADT7‐T served as the positive (+) and negative (–) controls, respectively. (E) GST pull‐down assay validating the interaction between SlCAT2 and SlAdBiL. Recombinant SlCAT2‐GST and SlAdBiL‐His proteins were expressed in and purified from *E. coli* strain BL21. The Input and pull‐down fractions were analyzed by immunoblotting using anti‐GST and anti‐His antibodies. (F) Time‐course transcript levels of *SlAdBiL* in tomato leaves following *T. absoluta* feeding (0, 3, 6, 12, and 24 h post‐feeding). Data are normalized to the 0‐h time point and the internal reference gene *SlActin* (n = 3). (G) In vitro ubiquitination of SlCAT2 by SlAdBiL. Recombinant SlAdBiL‐GST and SlCAT2‐His were expressed in *E. coli* strain BL21 and purified. In vitro ubiquitination assays were performed in the presence of HA‐tagged ubiquitin. Ubiquitinated proteins were detected by immunoblotting using anti‐HA, anti‐GST and anti‐His antibodies. (H) Effect of TaCRVP on SlAdBiL‐mediated ubiquitination of SlCAT2 in planta. (H‐i) *N. benthamiana* leaves were co‐infiltrated to transiently co‐express SlAdBiL‐GFP, SlCAT2‐Strep, Ub‐Flag, and either TaCRVP‐Myc or TaCRVP_Q51A_‐Myc. The IP and Input were subjected to immunoblot analysis using anti‐Strep, anti‐Flag, anti‐Myc, and anti‐GFP antibodies as indicated. (H‐ii) Relative ubiquitination level of SlCAT2 protein normalized to SlCAT2‐Strep (n = 3). (I) Effect of *SlAdBiL* silencing on the in vivo ubiquitination of SlCAT2. Total proteins from TRV2‐empty and TRV2‐*SlAdBiL* tomato plants were IP with an anti‐SlCAT2 antibody or control IgG, followed by immunoblotting with an anti‐Ub antibody. Input were subjected to immunoblot analysis using anti‐SlCAT2 and anti‐GAPDH antibodies. (J) Bioassays of *T. absoluta* larvae on *SlAdBiL*‐silenced tomato plants. (J‐i) Leaf consumption area and larval body weight of *T. absoluta* after feeding on TRV2‐empty and TRV2‐*SlAdBiL* tomato plants for 48 h (n = 18–20). (J‐ii) Representative images of the corresponding larvae described in (J‐i) after 48 h of feeding on TRV2‐*SlAdBiL* and TRV2‐empty tomato plants. Scale bars = 1 cm. Data are presented as mean ± SD. Multiple comparisons were analyzed using one‐way ANOVA followed by Tukey's HSD test (F, H). Different lowercase letters indicate statistically significant differences (*p* < 0.05). Statistical significance for was determined using two‐tailed Student's *t*‐tests (J) (ns, not significant; *, *p* < 0.05; **, *p* < 0.01; ***, *p* < 0.001; ****, *p* < 0.0001). The original images for all immunoblots shown in this figure are provided in Figure S4.

Given the observed decline in SlCAT2 protein levels following *T. absoluta* infestation (Figure 4B), we investigated whether its degradation is mediated by the 26S proteasome. Immunoblot analysis of WT leaves treated with the translation inhibitor CHX showed a rapid decrease in SlCAT2, which was largely rescued by co‐treatment with the proteasome inhibitor MG132 (Figure 5A). This indicates that SlCAT2 is degraded by the 26S proteasome pathway. To further determine if this degradation is ubiquitin‐dependent, we performed Co‐IP assays, successfully detecting ubiquitinated SlCAT2 in vivo with an anti‐Ub antibody, compared to the negative control IgG (Figure 5B). Together, these results establish that SlCAT2 undergoes continuous ubiquitin‐mediated degradation by the 26S proteasome.

To uncover the specific molecular mechanism driving the 26S proteasome‐mediated degradation of SlCAT2, we performed a Y2H screen to identify its interacting partners. We successfully isolated a RING‐type E3 ubiquitin ligase, designated SlAdBiL (Figure 5C). The direct physical interaction between SlCAT2 and SlAdBiL was initially confirmed by targeted Y2H assays and subsequently validated in vitro using a GST pull‐down assay (Figure 5D, E). Subsequent in vivo Co‐IP and LCI assays corroborated this physical interaction (Figure S19). Collectively, our results demonstrate that SlCAT2 directly interacts with the E3 ligase SlAdBiL. Finally, RT‐qPCR analysis revealed that herbivory significantly upregulated *SlAdBiL* transcript levels (Figure 5F).

Given that SlAdBiL is a RING‐type E3 ligase interacting with SlCAT2, we hypothesized that SlCAT2 is a ubiquitination substrate of SlAdBiL. We first confirmed the enzymatic activity of SlAdBiL by demonstrating its autoubiquitination in vitro in the presence of E1, E2, and ubiquitin (Figure 5G). Subsequently, in vitro ubiquitination assays with purified SlAdBiL‐GST and SlCAT2‐His showed that SlAdBiL directly ubiquitinates SlCAT2. This modification strictly required the presence of E1, E2, ubiquitin, and SlAdBiL, as no ubiquitination was detected when any single component was omitted. These results confirm SlAdBiL as the specific E3 ligase for SlCAT2 (Figure 5G).

To verify whether SlAdBiL mediates the ubiquitination of SlCAT2 in vivo, we transiently expressed SlCAT2‐Strep alone or in combination with SlAdBiL‐GFP in *N. benthamiana* leaves and assessed SlCAT2 ubiquitination by Co‐IP. Ubiquitinated SlCAT2‐Strep was detected even when expressed alone, likely due to the activity of endogenous E3 ligases in *N. benthamiana*. However, co‐expression with SlAdBiL‐GFP significantly enhanced the ubiquitination signal compared to the GFP control (Figure 5H). These results demonstrate that SlAdBiL mediates the ubiquitination of SlCAT2 in planta.

To further evaluate the physiological role of SlAdBiL in tomato resistance against *T. absoluta*, we used virus‐induced gene silencing (VIGS) to knock down *SlAdBiL* expression in planta. The silencing efficiency was confirmed by RT‐qPCR, showing a significant reduction of *SlAdBiL* transcripts in TRV2‐*SlAdBiL* plants compared to TRV2‐empty controls (Figure S20). Further in vivo ubiquitination assays confirmed that the endogenous ubiquitination level of SlCAT2 was markedly reduced in *SlAdBiL*‐silenced leaves (Figure 5I). Consistently, TRV2‐*SlAdBiL* plants exhibited significantly enhanced CAT activity and a substantial decrease in H_2_O_2_ accumulation and ROS burst relative to the control plants (Figure S21). We then performed insect bioassays to evaluate the impact of this compromised oxidative defense on herbivore performance. *T. absoluta* larvae feeding on TRV2‐*SlAdBiL* plants consumed significantly more leaf area and gained more weight compared to those on TRV2‐empty plants (Figure 5J). Collectively, these results indicate that SlAdBiL acts as a positive regulator of tomato immunity by promoting the proteasomal degradation of SlCAT2 to sustain the defensive ROS burst.

### 2.6 TaCRVP Enhances SlCAT2 Enzymatic Activity by Promoting Its Tetramerization

**FIGURE 6:**
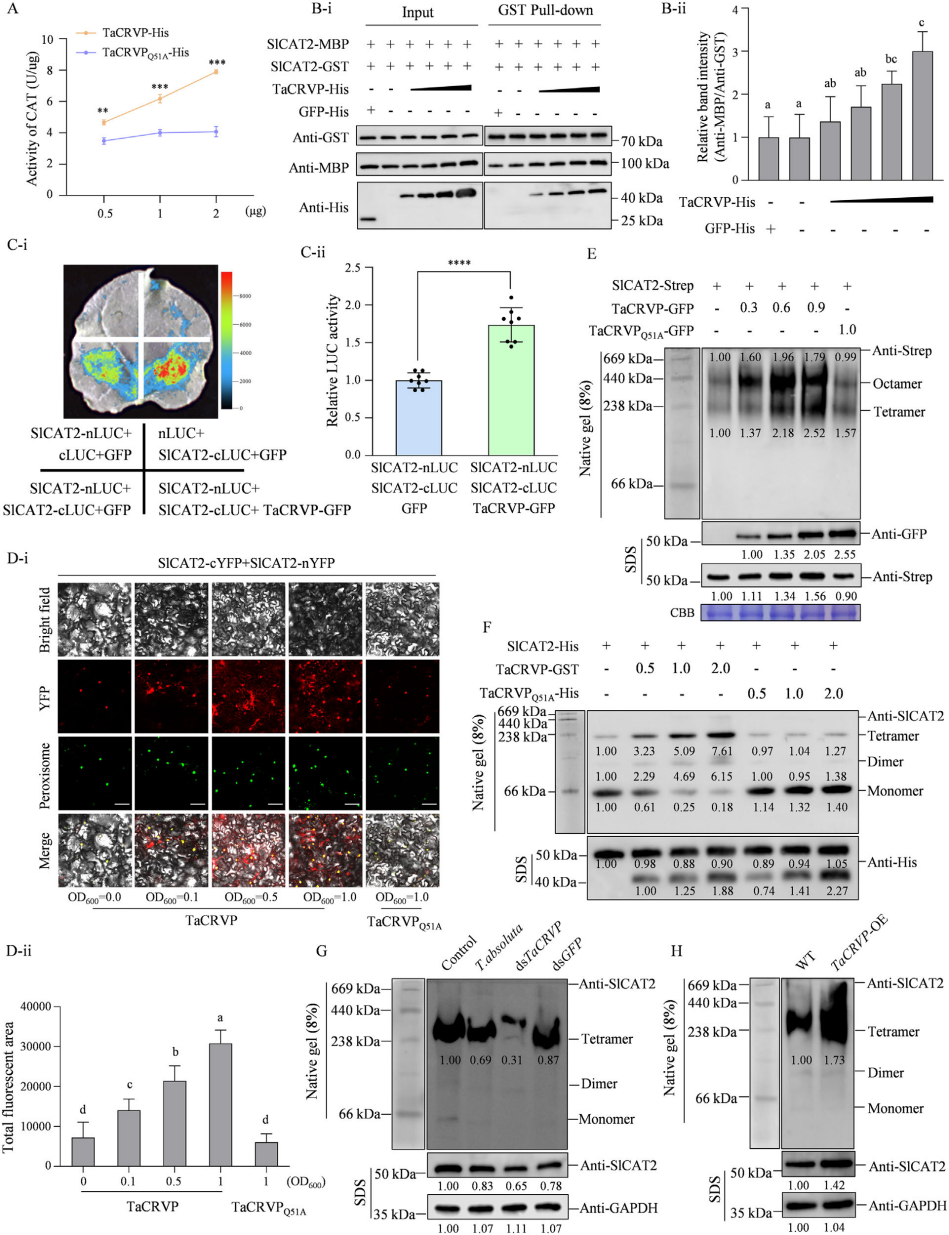
TaCRVP enhances SlCAT2 enzymatic activity by promoting its tetramerization. (A) In vitro catalase activity of purified SlCAT2‐His protein in the presence of recombinant TaCRVP‐His or the TaCRVP_Q51A_‐His. All recombinant proteins were expressed in *E. coli* strain BL21 and purified. CAT activity was measured by monitoring the decomposition of H_2_O_2_ using a catalase assay kit (n = 3). (B) TaCRVP promotes the self‐interaction of SlCAT2 in vitro. (B‐i) Representative immunoblots of the GST pull‐down assays. Recombinant SlCAT2‐GST, SlCAT2‐MBP, TaCRVP‐His and GFP‐His proteins were expressed in and purified from *E. coli* strain BL21. GST pull‐down assays were performed using SlCAT2‐GST and SlCAT2‐MBP in the presence of increasing concentrations of TaCRVP‐His. GFP‐His served as a negative control. The pull‐down fractions along with the Input samples were analyzed via immunoblotting using anti‐GST, anti‐MBP and anti‐His antibodies. (B‐ii) Relative quantification of the SlCAT2 self‐interaction. Band intensities were quantified using ImageJ software, and the signal of the prey protein (anti‐MBP) was normalized to that of the corresponding bait protein (anti‐GST) within the same lane (n = 3). (C) LCI assay confirming that TaCRVP enhances SlCAT2 self‐interaction. (C‐i) *N. benthamiana* leaves were co‐infiltrated with *A*. suspensions harboring the SlCAT2‐nLuc and SlCAT2‐cLuc fusion constructs, alongside either TaCRVP or a GFP control. Luminescence signals were captured at 48 h post‐infiltration after the application of D‐luciferin. Co‐expression of the target proteins with corresponding empty vectors served as negative controls. (C‐ii) Luciferase signals were quantified using ImageJ software, and the relative LUC activity of the control group was normalized to 1 (n = 8). (D) BiFC assay visualizing the dose‐dependent enhancement of SlCAT2 self‐interaction by TaCRVP. (D‐i) SlCAT2‐nYFP and SlCAT2‐cYFP were co‐expressed in *N. benthamiana* leaves with increasing concentrations of TaCRVP (OD_600_ = 0, 0.1, 0.5, 1.0) or TaCRVP_Q51A_ mutant. Scale bars = 50 µm. (D‐ii) Total fluorescence area intensity was quantified using ImageJ software (n = 3). (E) TaCRVP promotes the oligomerization of SlCAT2 in planta. Native PAGE analysis of the oligomeric state of SlCAT2‐Strep transiently co‐expressed with increasing concentrations of TaCRVP or the TaCRVP_Q51A_ mutant in *N. benthamiana* leaves. Total proteins were separated by native PAGE (8%) and subjected to immunoblot analysis. Corresponding basal protein expression levels were verified by SDS‐PAGE, with Coomassie Brilliant Blue (CBB) staining demonstrating equal protein loading. (F) TaCRVP promotes SlCAT2 oligomerization to enhance its catalase activity in vitro. A constant amount of purified SlCAT2‐His was incubated with increasing amounts (0, 0.5, 1.0, and 2.0 µg) of either TaCRVP‐His or TaCRVP_Q51A_‐His. The oligomeric states of SlCAT2 were analyzed by Native‐PAGE, demonstrating a progressive shift from monomers to dimeric and tetrameric forms with the addition of TaCRVP. SDS‐PAGE served as a loading control to confirm the constant total abundance of SlCAT2 and the corresponding gradient of TaCRVP proteins. (G) Native‐PAGE analysis of endogenous SlCAT2 oligomerization in tomato leaves under different infestation treatments. Assays were performed at 12 h post‐feeding by control (uninfested), wild‐type, ds*TaCRVP*‐, or ds*GFP*‐treated *T. absoluta* larvae. The basal accumulation of endogenous SlCAT2 was analyzed by SDS‐PAGE followed by immunoblotting. GAPDH served as the loading control. (H) Comparison of SlCAT2 oligomeric states in WT and *TaCRVP*‐OE tomato leaves by native PAGE. The basal accumulation of endogenous SlCAT2 was analyzed by SDS‐PAGE followed by immunoblotting. GAPDH served as the loading control. Data are presented as mean ± SD. Statistical significance was determined using two‐tailed Student's *t*‐tests (A, C) (ns, not significant; *, *p* < 0.05; **, *p* < 0.01; ***, *p* < 0.001; ****, *p* < 0.0001). Multiple comparisons were analyzed using one‐way ANOVA followed by Tukey's HSD test (B, D). Different lowercase letters indicate statistically significant differences based on ANOVA (*p* < 0.05). The original images for all immunoblots shown in this figure are provided in Figure S5.

ROS occupy a central node in plant immunity, functioning as direct defensive toxicants and vital secondary messengers [4, 24]. Within this complex network, H_2_O_2_ operates as a predominant signaling molecule due to its relative stability [25]. As the primary H_2_O_2_ scavenger, CAT dictates cellular redox homeostasis by rapidly catalyzing its decomposition [26]. To validate the H_2_O_2_‐scavenging capacity of SlCAT2, we expressed and purified a recombinant SlCAT2‐His fusion protein in *E. coli* BL21. H_2_O_2_ decomposition assays confirmed that SlCAT2‐His exhibited significant CAT activity compared with the the empty vector control (Figure S22). To delineate the biochemical consequences of the TaCRVP‐SlCAT2 interaction, we investigated whether the effector modulates host CAT activity in vitro. Following incubation of purified SlCAT2‐His with TaCRVP‐His or TaCRVP_Q51A_‐His, we found that TaCRVP significantly enhanced CAT activity of SlCAT2, an effect completely abolished by the Q51A mutation (Figure 6A). To substantiate these findings in planta, we transiently co‐expressed SlCAT2 with either TaCRVP or TaCRVP_Q51A_ in *N. benthamiana* leaves. Consistent with the in vitro data, TaCRVP, but not the mutant, significantly enhanced CAT activity (Figure S23A). Importantly, this effector‐driven enzymatic enhancement significantly promoted the feeding performance of *T. absoluta* larvae (Figure S23B).

Given that plant CATs function as hemoprotein tetramers [27, 28], we hypothesized that TaCRVP enhances enzymatic activity by promoting SlCAT2 holoenzyme assembly. To test this, we first verified that SlCAT2 monomers self‐interact via Y2H, GST pull‐down, Co‐IP, and LCI assays (Figure S24). Concurrently, analysis of truncated SlCAT2 mutants localized the self‐interaction domain to the catalase domain (Figure S25). Next, to determine whether TaCRVP actively facilitates this process, we conducted GST pull‐down, LCI, Co‐IP and assays. Strikingly, these results consistently showed that increasing concentrations of TaCRVP progressively enhanced the SlCAT2 self‐interaction (Figures 6B, C; Figure S26). This effect was visually corroborated by bimolecular fluorescence complementation (BiFC) assays in planta, which showed a significant, concentration‐dependent increase in fluorescent puncta upon TaCRVP co‐expression. Crucially, this promotional effect was completely abolished when the interaction‐deficient TaCRVP_Q51A_ mutant was co‐expressed (Figure 6D). Collectively, these results demonstrate that TaCRVP specifically promotes the self‐association of SlCAT2 monomers.

To further investigate this assembly process, we co‐expressed SlCAT2 with either TaCRVP or the TaCRVP_Q51A_ mutant in *N. benthamiana* and assessed the formation of protein complexes via native‐PAGE. Strikingly, SlCAT2 expressed alone assembled into distinct high‐molecular‐weight complexes of approximately 228 and 456 kDa, consistent with putative tetramers and octamers of the 57 kDa monomer (Figure 6E). Notably, co‐expression with TaCRVP triggered a clear dose‐dependent increase in these SlCAT2 complexes, the TaCRVP_Q51A_ mutant failed to do so (Figure 6E). However, we noted that TaCRVP co‐expression in *N. benthamiana* concurrently elevated total SlCAT2 protein levels (Figure 6E). To strictly exclude the possibility that the observed oligomer enrichment was merely an indirect consequence of this increased protein abundance, and to explicitly rule out non‐specific aggregation of the proteins, we performed an in vitro titration assay. We established the baseline oligomeric state using a constant amount of purified SlCAT2‐His. Native‐PAGE revealed that SlCAT2 alone successfully forms dimeric and tetrameric structures alongside monomers (Figure 6F, lane 1). Upon co‐incubation, increasing concentrations of TaCRVP‐His progressively reduced the SlCAT2 monomer band and concomitantly enriched the active tetrameric form without altering total SlCAT2 abundance (Figure 6F). Finally, we examined the oligomeric status of endogenous SlCAT2 in tomato leaves. As anticipated, SlCAT2 predominantly existed as a tetramer (Figure 6G). Notably, SlCAT2 tetramer accumulation was significantly reduced in tomato plants fed upon by *TaCRVP*‐silenced *T. absoluta*, whereas *TaCRVP*‐OE plants exhibited increased tetramer levels relative to WT (Figure 6G, H). Collectively, these results demonstrate that TaCRVP actively promotes the formation of SlCAT2 tetramers to disrupt plant oxidative defenses.

### 2.7 TaCRVP Competitively Shields SlCAT2 From SlAdBiL‐Mediated Proteasomal Degradation

Given that SlCAT2 undergoes continuous ubiquitin‐mediated degradation by the 26S proteasome, and that TaCRVP enhances SlCAT2 protein accumulation, we profiled the SlCAT2 protein accumulation during *T. absoluta* infestation in WT and *TaCRVP*‐OE plants. Although herbivory triggered a visible decline in SlCAT2 abundance across all genotypes, the basal and post‐infestation protein levels remained constitutively higher in the *TaCRVP*‐OE lines (Figure 5A). Furthermore, the rapid CHX‐induced degradation of SlCAT2 observed in WT plants was significantly delayed in the presence of TaCRVP (Figure 5A). Since *SlCAT2* transcript levels showed no significant difference between WT and *TaCRVP*‐OE plants (Figure S27), we reasoned that TaCRVP actively stabilizes SlCAT2 by shielding it from ubiquitination. To confirm this, we assessed in vivo SlCAT2 ubiquitination levels via Co‐IP. Strikingly, the polyubiquitination signal of SlCAT2 was substantially reduced in the *TaCRVP*‐OE background compared to the WT (Figure 5B). Together, these in planta results confirmed that TaCRVP stabilizes SlCAT2 by inhibiting its ubiquitin‐proteasome‐dependent degradation.

To determine whether TaCRVP interferes with SlAdBiL‐mediated degradation of SlCAT2, we co‐expressed SlCAT2‐Strep and SlAdBiL‐GFP alongside either TaCRVP‐Myc or TaCRVP_Q51A_‐Myc in *N. benthamiana* leaves. Co‐IP analysis revealed that TaCRVP‐Myc, but notably not the TaCRVP_Q51A_‐Myc mutant, significantly suppressed the SlAdBiL‐mediated polyubiquitination of SlCAT2 (Figure 5H). Based on these results, we propose that the direct physical binding of TaCRVP sterically shields SlCAT2, thereby competitively inhibiting its interaction with and subsequent ubiquitination by the SlAdBiL.

**FIGURE 7:**
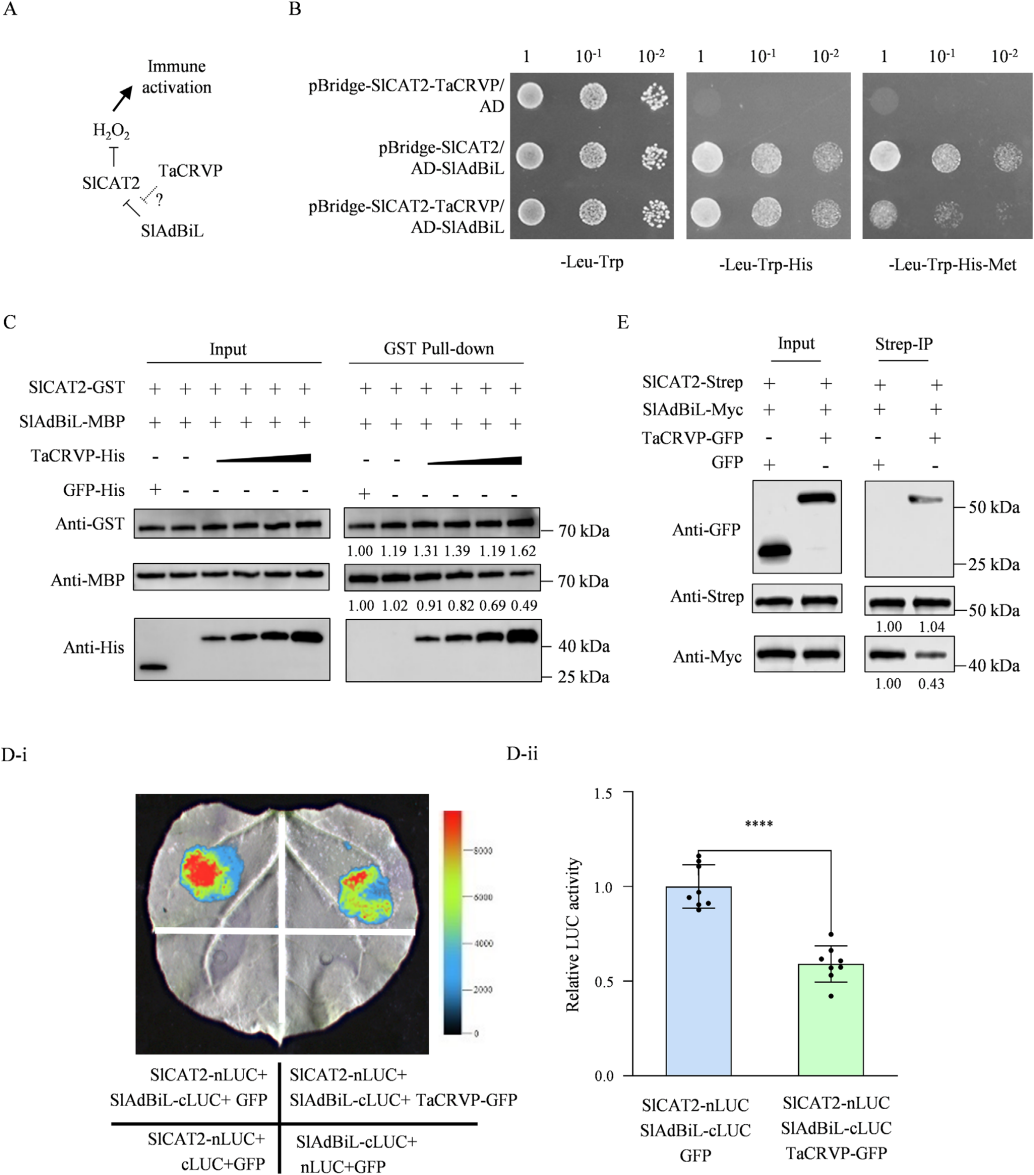
TaCRVP competitively shields SlCAT2 from SlAdBiL‐mediated proteasomal degradation. (A) Schematic illustration showing the interplay among TaCRVP, SlCAT2 and SlAdBiL. (B) Y3H assay demonstrating that TaCRVP competitively inhibits the binding between SlAdBiL‐SlCAT2. Yeast cells co‐expressing AD‐SlAdBiL and the pBridge‐SlCAT2‐TaCRVP construct (in which TaCRVP expression is strictly controlled by a methionine‐repressible promoter) were spotted onto selective media. Cell growth on SD/–Trp/–Leu/–His medium in the presence of methionine (+Met; TaCRVP repressed) or absence of methionine (–Met; TaCRVP expressed) enables the direct visual assessment of competitive inhibition. Co‐transformations with pBridge‐SlCAT2‐TaCRVP plus empty pGADT7, and pBridge‐SlCAT2 plus AD‐SlAdBiL, served as the negative and positive controls, respectively. Serially diluted yeast cultures (1, 10^−^^1^, and 10^−^^2^) are shown. (C) In vitro GST pull‐down assays revealing the competitive inhibition of the SlCAT2‐SlAdBiL interaction by TaCRVP. Recombinant SlCAT2‐GST, SlAdBiL‐MBP, TaCRVP‐His, and GFP‐His proteins were expressed in and purified from *E. coli* strain BL21. GST pull‐down assays were performed using SlCAT2‐GST and SlAdBiL‐MBP in the presence of increasing concentrations of TaCRVP‐His. GFP‐His served as a negative control. The pull‐down fractions along with the Input samples were analyzed via immunoblotting using anti‐GST, anti‐MBP, and anti‐His antibodies. (D) LCI assay confirming that TaCRVP disrupts the SlCAT2‐SlAdBiL interaction. (D‐i) *N. benthamiana* leaves were co‐infiltrated with *A. tumefaciens* suspensions harboring the SlCAT2‐nLuc and SlCAT2‐cLuc fusion constructs, alongside either TaCRVP or a GFP control. Luminescence signals were captured at 48 h post‐infiltration after the application of D‐luciferin. Co‐expression of the target proteins with corresponding empty vectors served as negative controls. (D‐ii) Luciferase signals were quantified using ImageJ software, and the relative LUC activity of the control group was normalized to 1 (n = 8). (E) Co‐IP assay confirming that TaCRVP disrupts the SlCAT2‐SlAdBiL interaction in planta. *N. benthamiana* leaves were agroinfiltrated to transiently co‐express SlCAT2‐Strep and SlAdBiL‐Myc, in the presence of either TaCRVP‐GFP or a free GFP control. Input was subjected to IP using anti‐Strep agarose beads. Both the Input and IP fractions were analyzed by immunoblotting using anti‐Strep, anti‐Myc, and anti‐GFP antibodies to detect the respective proteins. GFP served as a negative control. Data are presented as mean ± SD. Statistical significance was determined using two‐tailed Student's *t*‐tests (ns, not significant; *, *p* < 0.05; **, *p* < 0.01; ***, *p* < 0.001; ****, *p* < 0.0001). The original images for all immunoblots shown in this figure are provided in Figure S6.

To establish whether TaCRVP competitively interferes with the SlCAT2‐SlAdBiL interaction (Figure 7A), we used a yeast three‐hybrid (Y3H) system. In the presence of methionine (repressing TaCRVP expression), yeast grew on selective medium, confirming the SlCAT2‐SlAdBiL interaction. When TaCRVP expression was induced (in the absence of methionine), yeast growth was strongly inhibited, indicating that TaCRVP disrupts this interaction (Figure 7B). This finding was corroborated by in vitro competitive pull‐down assays, where increasing concentrations of TaCRVP‐His progressively reduced the amount of SlAdBiL‐MBP pulled down by SlCAT2‐GST, demonstrating a clear dose‐dependent competition (Figure 7C). Furthermore, Co‐IP and LCI assays in *N. benthamiana* consistently showed that TaCRVP significantly attenuates the association between SlCAT2 and SlAdBiL (Figure 7D, E). Collectively, these results indicate that TaCRVP enhances SlCAT2 protein stability by competing with SlAdBiL for binding to SlCAT2, thereby inhibiting SlAdBiL‐mediated ubiquitination and degradation.

## 3 Discussion

While the suppression of host immunity by salivary effectors is a well‐recognized strategy in plant‐herbivore interactions, the molecular mechanisms deployed by lepidopteran pests remain incompletely characterized. In this study, we uncover a a novel counter‐defense strategy utilized by *T. absoluta*. We identify TaCRVP, a venom‐like effector belonging to the widespread CAP protein superfamily [29, 30, 31], and demonstrate that it actively subverts plant immunity by directly targeting the the host ROS‐scavenging enzyme SlCAT2. Unlike previously reported effectors that broadly modulate redox states, TaCRVP employs a dual‐hijacking mechanism whereby it activates host catalase by promoting its oligomerization, while post‐translationally inhibiting its ubiquitination. This dual‐layered counter‐strategy not only establishes TaCRVP as a critical salivary effector, but also reveals how an insect can comprehensively manipulate host CAT to suppress the H_2_O_2_ burst and facilitate sustained herbivory.

Upon herbivory, plants rapidly generate a ROS burst as an early signal. While the roles of CATs in regulating ROS dynamics against microbial pathogens and abiotic stresses have been extensively studied [32, 33, 34], their specific dynamic regulation during insect herbivory remains poorly characterized. Our findings demonstrate that SlCAT2 acts as a major negative regulator of herbivore resistance. We discovered that tomato plants suppress SlCAT2 at both the transcriptional and protein levels upon *T. absoluta* infestation to promote ROS accumulation. Crucially, the endogenous E3 ubiquitin ligase SlAdBiL physically interacts with SlCAT2, mediating its ubiquitination and degradation. Consequently, the SlAdBiL‐SlCAT2 module functions as a key regulator, ensuring the sustained ROS accumulation necessary for downstream defense signaling.

To overcome this host‐mediated degradation, *T. absoluta* secretes TaCRVP, which stabilizes SlCAT2 to restore cellular redox homeostasis. Recent studies have reported diverse pathogen and insect effectors that suppress ROS, such as *Phytophthora sojae* PsCRN63 and PsCRN115 [34], *Pepino mosaic* virus TGBp1 [35], and various hemipteran salivary proteins [14, 36, 37, 38]. However, the molecular mechanisms by which these effectors suppress the host ROS burst remain poorly understood. Here, we demonstrate that TaCRVP directly interacts with host catalase to modulate its activity and stability. Similar to how the wheat regulator TaWD40‐4B.1 promotes TaCAT3 oligomerization [39], TaCRVP interacts with SlCAT2 to promote its assembly into highly active tetramers, enhancing its enzymatic activity. Furthermore, TaCRVP competitively inhibits the SlCAT2‐SlAdBiL interaction, protecting SlCAT2 from SlAdBiL‐mediated ubiquitination and degradation. Beyond SlCAT2, the ability of TaCRVP to target functionally redundant host paralogs (SlCAT1 and SlCAT3) as well as conserved CATs in related species (e.g., tobacco) underscores an evolutionarily convergent strategy to maintain effective ROS suppression across different host plants.

Given the role of ROS as key signaling molecules, the TaCRVP‐mediated suppression of ROS accumulation effectively compromises downstream systemic defense. Previous studies have shown that *T. absoluta* feeding elicits extensive defense networks, including MAPK cascades and the massive accumulation of defensive secondary metabolites [40]. Notably, our integrated transcriptomic and metabolomic analyses demonstrate that TaCRVP overexpression disrupts these defense networks. Specifically, we found a significant suppression of the linoleic and α‐linolenic acid metabolism pathways, which provide the essential upstream precursors for JA biosynthesis [41, 42]. Consequently, the accumulation of downstream defensive compounds, including phenolics, flavonoids, and lignin, was significantly reduced. These metabolic profiles demonstrate that TaCRVP impairs the activation of JA‐dependent defenses by attenuating the early H_2_O_2_ burst, thereby increasing host susceptibility to *T. absoluta*.

**FIGURE 8:**
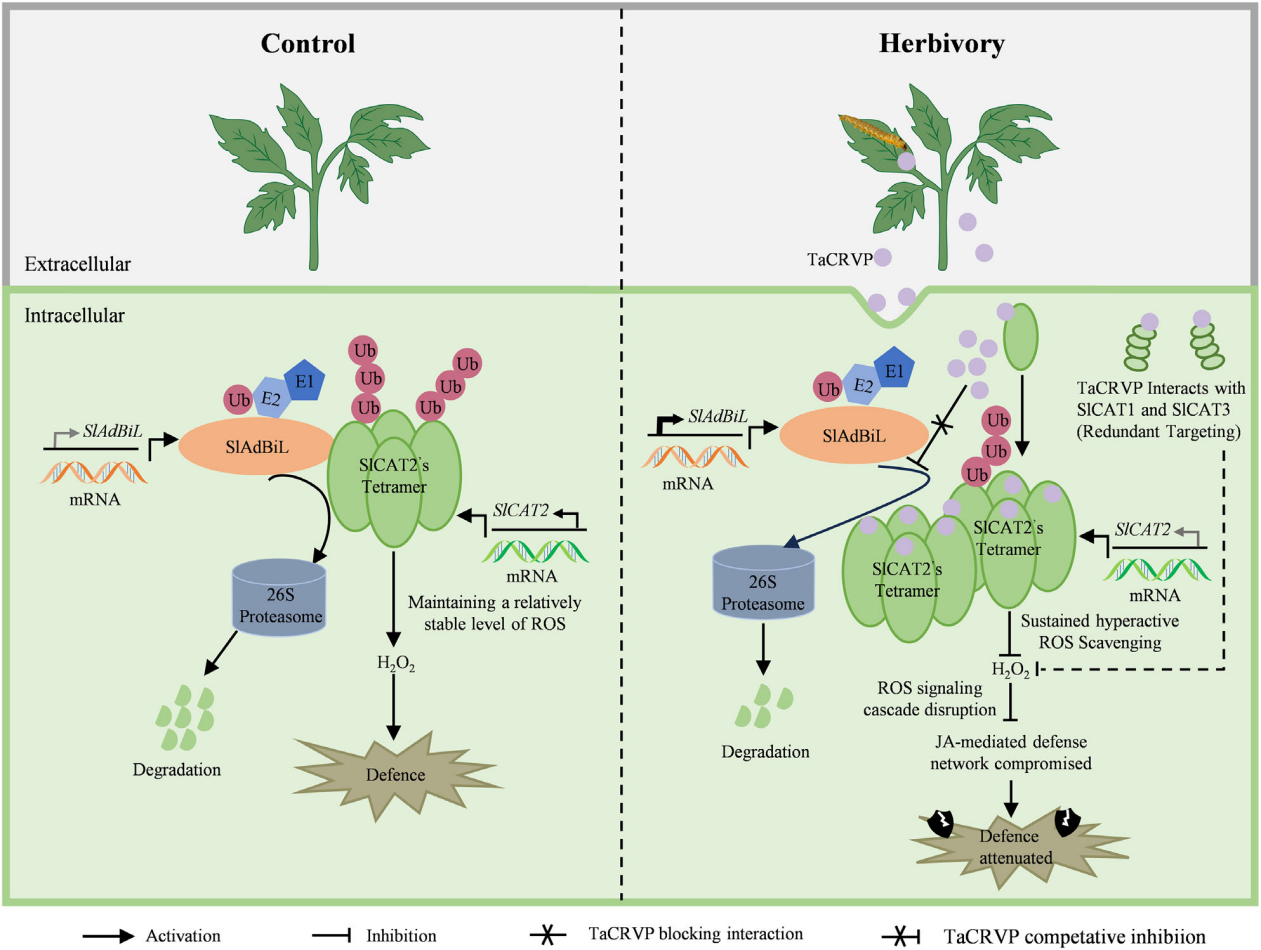
Working model illustrating the subversion of tomato oxidative defenses by the *T. absoluta* salivary effector TaCRVP. Upon herbivory, tomato plants activate an oxidative defense by downregulating *SlCAT2* transcription while upregulating the *SlAdBiL* to target the SlCAT2 for 26S proteasome‐dependent degradation. To counteract this immune response, *T. absoluta* secretes TaCRVP into host cells, where it manipulates SlCAT2 through a dual mechanism. First, TaCRVP interacts with SlCAT2 to promote its tetramerization and significantly enhance its enzymatic activity. Concurrently, it competitively blocks the SlCAT2‐SlAdBiL interaction, protecting the SlCAT2 from SlAdBiL‐mediated ubiquitination and degradation. Furthermore, TaCRVP targets the functionally redundant paralogs SlCAT1 and SlCAT3 to further suppress H_2_O_2_ accumulation. Ultimately, this disruption of ROS signaling suppresses the downstream JA‐mediated defense network, thereby facilitating extensive insect infestation.

Based on these findings, we propose a working model illustrating how TaCRVP manipulates the SlCAT2‐SlAdBiL module to subvert H_2_O_2_ homeostasis and plant immunity (Figure 8). In the absence of herbivory, this module maintains baseline cellular redox balance through tightly regulated SlCAT2. Upon perception of *T. absoluta* feeding, the host rapidly activates an oxidative defense by downregulating *SlCAT2* transcription while upregulating the E3 ligase *SlAdBiL*. This upregulated SlAdBiL then physically interacts with SlCAT2, mediating the ubiquitination and proteasomal degradation of the SlCAT2, thereby sustaining the H_2_O_2_ burst for plant immunity. However, *T. absoluta* secretes the salivary effector TaCRVP to subvert this oxidative burst. First, TaCRVP interacts with SlCAT2 to promote its tetramerization and significantly enhance its enzymatic activity. Concurrently, it competitively blocks the SlCAT2‐SlAdBiL interaction, protecting the SlCAT2 from SlAdBiL‐mediated ubiquitination and degradation. Furthermore, TaCRVP targets the functionally redundant paralogs SlCAT1 and SlCAT3 to further suppress H_2_O_2_ accumulation. Ultimately, this disruption of ROS signaling suppresses the downstream JA‐mediated defense network, thereby facilitating extensive insect herbivory.

Future studies are required to elucidate the cellular entry route of TaCRVP [43]. the upstream transcriptional regulation of the SlCAT2‐SlAdBiL module under herbivory stress, and the crosstalk between H_2_O_2_ dynamics and downstream JA‐mediated defense networks [44]. Because the complete knockout of SlCAT2 causes severe fitness costs due to excessive basal ROS, utilizing precise gene editing (e.g., CRISPR‐Cas9) to mutate the TaCRVP‐binding interface on SlCAT2 could uncouple this growth‐defense trade‐off. This targeted approach would abolish the effector‐target interaction without disrupting the plant physiological redox balance, offering a rational strategy for breeding crops with enhanced resistance to *T. absoluta*.

## 4 Experimental Section

### 4.1 Plant Growth and Insect Rearing

Tomato (*Solanum lycopersicum*) and *N. benthamiana* plants were grown in a soil‐perlite‐vermiculite mix (3:1:1, v/v/v) under controlled conditions (25 ± 1°C, 16‐h light/8‐h dark, 65 ± 5% relative humidity). *N. benthamiana* at the four‐leaf stage was utilized for *A. tumefaciens*‐mediated transient expression.

The *T. absoluta* population, originally collected from Yunnan Province, China, was maintained at Fujian Agriculture and Forestry University (Fuzhou, China) under identical climatic conditions. Larvae were reared on either tomato plants or a synthetic artificial diet. The diet (per 1000 mL ultrapure water) comprised 30 g wheat germ, 8 g cellulose, 45 g saccharose, 0.2 g wesson salts, 42 g casein, 4.6 g ascorbic acid, 1.4 g sorbic acid, 1.4 g tetracycline, and 32 g agar. The mixture was homogenized, heated to completely dissolve the agar, and dispensed into sterile containers.

### 4.2 *T. absoluta* Saliva Collection and LC‐MS/MS Proteomic Analysis

OSs were collected from approximately 300 third‐ and fourth‐instar *T. absoluta* larvae (reared on fresh tomato leaves) via tactile stimulation using a pipette tip. Pooled OSs (∼200 µL) were centrifuged (12,000 × *g*, 10 min, 4°C) to remove debris. Samples were flash‐frozen in liquid nitrogen, ground. and lysed in SDT buffer (4% SDS, 100 mM Tris‐HCl, pH 7.6, 1% DTT) via ultrasonication (5 min, ice‐water bath). After centrifugation (12,000 × *g*, 15 min, 4°C), supernatant were denatured at 95°C for 10 min, alkylated with iodoacetamide in the dark for 1 h, and precipitated with four volumes of cold acetone at −20°C for at least 30 min. Protein pellets were washed, air‐dried, and reconstituted in dissolution buffer (8 m urea, 100 mM Tris‐HCl, pH 8.0). Protein concentration was determined using a bicinchoninic acid (BCA) protein assay.

A total of 20 µg of proteins were separated by 12% SDS‐PAGE and stained with Coomassie Brilliant Blue R‐250. Excised bands underwent in‐gel digestion overnight at 37°C using 1 µg of sequencing‐grade modified trypsin (Promega, USA; V5111) in 100 mM triethylammonium bicarbonate (TEAB) buffer. Peptides were extracted using acetonitrile (ACN) and 0.1% formic acid (FA), pooled, lyophilized and desalted using a C18 spin column (Thermo Fisher Scientific, USA). Desalted peptides (1 µg) in 0.1% FA were analyzed on a Vanquish Neo UHPLC system coupled to an Orbitrap Astral mass spectrometer (Thermo Fisher Scientific, USA). Separation was performed on a C18 analytical column (150 µm × 15 cm, 2 µm) using a 13.7‐min gradient of 4% to 55% mobile phase B (0.1% FA in 80% ACN) at varying flow rates (1.5–2.5 µL/min). Data‐independent acquisition (DIA) MS1 full scans were acquired at 240,000 resolution (*m/z* 380–980, AGC target 500%). MS2 spectra were acquired using 300 precursor isolation windows (2 Th) at 80,000 resolution (*m/z* 150–2000, normalized collision energy 25%, maximum injection time 3 ms).

Raw DIA data were processed using Spectronaut (Biognosys, Switzerland). against the UniProt Lepidoptera database (Figure S28). Parameters included trypsin specificity (≤ 2 missed cleavages), carbamidomethylation of cysteine (fixed modification), and methionine oxidation/N‐terminal acetylation (variable modifications). Mass tolerances were set to 10 ppm (precursor) and 0.02 Da (fragment). The false discovery rate (FDR) was strictly controlled at < 1% at both precursor and protein levels.

### 4.3 Sequence Analysis

The signal peptide and transmembrane domains of TaCRVP were predicted using the SignalP 6.0 and TMHMM 2.0 servers, respectively. The conserved domains of TaCRVP, SlCAT2, and SlAdBiL were annotated via the InterPro database. Homologous proteins were identified via BLASTP searches against the NCBI non‐redundant (nr) database. Specifically, the SlCAT2 sequence was used to identify homologs in *N. benthamiana*.

### 4.4 RNA Extraction and RT‐qPCR Analysis

To assess *TaCRVP* expression, *T. absoluta* samples were collected across developmental stages (first to fourth instar), tissues (gut, fat body, salivary glands, head, and integument dissected from third‐instar larvae in cold PBS), and feeding time‐courses (0, 3, 6, 12, and 24 h post‐transfer to tomato or artificial diet following a 12‐h starvation). For plant gene expression analyses, leaves adjacent to feeding sites (along with uninfested controls) from six‐week‐old WT and *TaCRVP*‐OE tomato plants were harvested after 2 h of larval infestation. Time‐course leaf samples were similarly collected for *SlAdBiL* (0, 3, 6, 12, and 24 h post‐infestation) and *SlCAT2* (which included additional early time points at 0.25, 0.5, and 1 h). All collected insect and plant samples were immediately flash‐frozen in liquid nitrogen and stored at −80°C.

Total RNA extraction using TRIpure Reagent (Herui Biotech, Fujian, China; HRN0142) and cDNA synthesis using the Hifair AdvanceFast first Strand cDNA Synthesis Kit (Yeasen Biotech, Shanghai, China; 11149ES60), were performed according to the manufacturers' instructions. Quantitative real‐time PCR (RT‐qPCR) was conducted using 2 × Universal SYBR RT‐qPCR Mix (Magen Biotech, Guangzhou, China; MD70101) on a CFX Connect System (Bio‐Rad, Hercules, CA, USA). Relative expression levels were calculated using the 2^−ΔΔCt^ method, normalized to *EF1α* (for *T. absoluta*) or *SlActin* (for tomato). All assays included three independent biological and three technical replicates. Primer sequences are listed in Table S1.

### 4.5 Complementary DNA (cDNA) Cloning

cDNA was synthesized from total RNA using the PrimeScript first strand cDNA Synthesis Kit (Takara Bio Inc., Shiga, Japan; R050A). The full‑length coding sequence of *TaCRVP*, *SlCAT2*/*SlAdBiL*, and Nb01049 were amplified from *T. absoluta*, *S. lycopersicum*, and *N. benthamiana* cDNA, respectively, using PrimeSTAR GXL DNA Polymerase (Takara Bio Inc., 6110A). The PCR products were gel‐purified (Sangon Biotech, Shanghai, China; B518131), A‐tailing using *Taq* DNA polymerase, ligated into the pMD19‐T vector (Takara Bio Inc., 3271), and sequence‐verified. All primer sequences are listed in Table S1

### 4.6 Transgenic Plant Generation and Characterization

All genetic constructs, including the TaCRVP‐Flag and SlCAT2‐Myc overexpression vectors and the CRISPR/Cas9 components for generating the SlCAT2‐KN mutant, were transformed into the Micro‐Tom (MT) tomato background via *Agrobacterium*‐mediated transformation by BIORUN BIOSCIENCES Co. Ltd. (Wuhan, China). Primary (T0) transformants were selected on hygromycinand and verified by PCR. For the SlCAT2‐KN lines, targeted genomic regions were Sanger‐sequenced to confirm editing events. To obtain homozygous T1 individuals for subsequent experiments, *SlCAT2*‐OE and *TaCRVP*‐OE lines were screened via RT‐qPCR and anti‐Flag (Sigma‐Aldrich, USA; F1804) immunoblotting, respectively. Homozygous *SlCAT2*‐KN mutants were identified by PCR genotyping and Sanger sequencing. Relevant primer sequences are listed in Table S1.

### 4.7 Yeast Signal Sequence Trap System

The yeast signal sequence trap assay was performed as previously described [45]. The coding sequences for *TaCRVP* ^ΔSP^ (lacking its signal peptide) and *TaCRVP* ^SP^ (signal peptide only) were cloned into the pSUC2 vector. These constructs, along with positive (*Avr1b* ^SP^) and negative (empty pSUC2) controls, were transformed into the *S. cerevisiae* strain YTK12. Transformed were selected on CMD‐W plates and patched onto both YPRAA (containing 2 µg/mL antimycin A) and CMD‐W media. For functional assessment, positive colonies were cultured in liquid medium, harvested and incubated with 10% sucrose and 1% TTC at 35°C. Colorimetric changes were photographed. Primer sequences are listed in Table S1.

### 4.8 H_2_O_2_ Measurement and DAB Staining

To investigate the effect of TaCRVP on H_2_O_2_ accumulation during *T. absoluta* infestation, leaf tissues adjacent to feeding sites, alongside uninfested controls, were collected at 0, 3, 6, 12, and 24 h post‐infestation by wild‐type, ds*TaCRVP*‐treated, or ds*GFP*‐treated larvae (n = 8 biological replicates). Additional H_2_O_2_ quantifications (n = 3 biological replicates per treatment/genotype) were performed on transgenic tomato lines. Specifically, leaves from WT and *TaCRVP*‐OE plants were collected at 0, 3, and 6 h post‐infestation. To investigate the role of the SlCAT2 genotype, WT, *SlCAT2*‐KN, and *SlCAT2*‐OE plants were infested with ds*TaCRVP*‐ or ds*GFP*‐treated larvae, and tissues were collected after a 3‐h feeding period. For all experiments, fresh leaves were ground in liquid nitrogen, and H_2_O_2_ concentrations were quantified using an H_2_O_2_ Assay Kit (Solarbio Science & Technology, Beijing, China; BC3590) following the manufacturer's protocol.

To assess the effects of TaCRVP and its mutant variants on the flg22‐induced ROS burst, transient co‐expression assays were performed in *N. benthamiana*. Coding sequences of *TaCRVP*, the point mutant *TaCRVP*_Q51A_, and truncation mutants *TaCRVP*m1–m3 were cloned into pGD vectors and transformed into *A. tumefaciens* strain GV3101. Bacterial suspensions mixed with flg22 were co‐infiltrated into *N. benthamiana* leaves. Leaves co‐infiltrated with an empty vector and flg22 served as negative controls. At 2 days post‐infiltration, the leaves were either harvested for H_2_O_2_ quantification or subjected to DAB staining following a previously described method with slight modifications [36]. For DAB staining, leaves were incubated in a 1 mg/mL DAB solution (Sigma‐Aldrich, D7304) at 65°C for 4 h, rinsed, destained in absolute ethanol until cleared, and photographed under natural light. All primer sequences used for plasmid construction are listed in Table S1.

### 4.9 ROS Assay

To assess the impact of TaCRVP and its variants on the ROS burst, transient expression was performed by infiltrating *N. benthamiana* leaves with *Agrobacterium* strains harboring *TaCRVP*, the point mutant *TaCRVP*_Q51A_, truncation mutants *TaCRVP*m1–m3, or an empty vector expressing *GFP* (negative control). At 48 h post‐infiltration, leaf discs (4–5 mm in diameter) were excised. For stable transgenic assays, identical leaf discs were collected from WT, *TaCRVP*‐OE, *SlCAT2*‐OE, and *SlCAT2*‐KN tomato plants. To eliminate wounding‐induced ROS background, individual discs were incubated overnight (12–16 h) at room temperature in 96‐well plates containing 100 µL of sterile double‐distilled water (ddH_2_O) per well. Following incubation, the water was removed and immediately replaced with100 µL of a reaction solution containing 10 µg/mL horseradish peroxidase (Sigma‐Aldrich, P8375), 100 nM flg22 elicitor (GenScript Biotech, Nanjing, China; RP19986), and either 50 µM luminol or 5 µM L‐012 (Sigma‐Aldrich, A8511, SML2236). Luminescence was continuously recorded every 1.5 min using a GloMax Discover GM3000 Multimode Microplate Reader (Promega, USA). ROS production dynamics were represented as relative light units (RLU).

### 4.10 SlCAT2 Enzyme Activity Assay

Total CAT enzyme activity was evaluated under three distinct experimental conditions. In the in vivo tomato assay, WT, *SlCAT2*‐KN, and *SlCAT2*‐OE tomato plants were infested with ds*TaCRVP*‐ or ds*GFP*‐treated *T. absoluta* larvae, and leaf tissues adjacent to feeding sites were collected after a 3‐h feeding period. For the in vitro recombinant protein assay TaCRVP and TaCRVP_Q51A_ (in pET‐28b) were expressed in *E. coli* BL21 and affinity‐purified. Purified SlCAT2‐His (2 µg) was co‐incubated with varying amounts (0.5, 1, and 2 µg) of His‐TaCRVP or His‐TaCRVP_Q51A_ in 30 µL reaction buffer (50 mM Tris‐HCl, pH 7.5, 150 mM NaCl, 1 mM DTT) for 1 h at 25°C. In the in planta transient assay, *SlCAT2* (in pGD) was expressed in *N. benthamiana* leaves via *A. tumefaciens* (OD600 = 0.5) either alone or co‐expressed with *TaCRVP* or *TaCRVP*_Q51A_ (in pGD), and leaf tissues were harvested 2 days post‐infiltration. For all assays, samples were ground in liquid nitrogen (where applicable) and CAT enzyme activity was quantified using a Catalase Assay Kit (Solarbio Science & Technology, BC4785) according to the manufacturer's protocol. All primer sequences used for plasmid construction are listed in Table S1.

### 4.11 RNAi‑Mediated Knockdown of *TaCRVP* in *T. absoluta* Via ds*TaCRVP* Soaking

To generate dsRNA, *TaCRVP* and *GFP* (control) fragments flanked by T7 promoters were amplified and subjected to in vitro transcription using the T7 RiboMAX Express RNAi System (Promega, P1700). Fluorescein‐labeled dsRNA was concurrently synthesized using the HyperScribe T7 High Yield Fluorescein RNA Labeling Kit (APExBIO, USA; K1060). Following purification and quantification, second‐instar *T. absoluta* larvae were soaked in the respective dsRNA solutions (2000 ng/µL) for 10 min and then transferred to fresh tomato leaves [46]. To verify systemic delivery, larvae treated with fluorescein‐labeled dsRNA were collected at 12 h post‐soaking. Fluorescent signals in intact larvae, as well as dissected salivary glands and midguts, were then visualized using a fluorescence microscope. To evaluate RNAi efficiency and temporal silencing dynamics, larvae soaked in ds*TaCRVP* or ds*GFP* were collected at 0 (untreated control), 6, 12, 24, 48, 72, 96, and 120 h post‐treatment, and relative *TaCRVP* transcript levels were determined via RT‐qPCR. All primer sequences used for dsRNA synthesis are listed in Table S1.

### 4.12 T. absoluta Bioassays

To systematically evaluate *T. absoluta* feeding performance across various genetic backgrounds, bioassays were conducted using several distinct plant and insect setups. For transient expression assays, *N. benthamiana* leaves were infiltrated with *A. tumefaciens* harboring *TaCRVP* (alone or co‐expressed with *SlCAT2*), the point mutant *TaCRVP*_Q51A_ (co‐expressed with *SlCAT2*), or an empty vector, and were utilized for insect feeding at 2 days post‐infiltration. For stable transgenic and VIGS tomato assays, fresh leaves were collected from WT, *TaCRVP*‐OE, TRV2‐empty, and TRV2‐*SlAdBiL* plants. To investigate the influence of the *SlCAT2* genotype on the feeding performance of dsRNA‐treated larvae, larvae were soaked in ds*TaCRVP* or ds*GFP* solutions for exactly 10 min and maintained on fresh tomato leaves for 72 h before being transferred to WT, *SlCAT2*‐KN, or *SlCAT2*‐OE tomato leaves. For all the aforementioned bioassays, twenty third‐instar larvae from the corresponding treatments were individually transferred onto the respective fresh leaves. Following a strict 48‐h feeding period, larval performance was comprehensively evaluated by quantifying the leaf consumption area and individual larval body weight.

### 4.13 Hormone Measurement

To investigate phytohormone accumulation dynamics during *T. absoluta* infestation, leaf tissues adjacent to the feeding sites (alongside uninfested controls), were collected under two experimental conditions. In the insect‐treatment assay, WT tomato plants were infested with wild‐type, ds*TaCRVP*‐, or ds*GFP*‐treated larvae, and samples were collected at 0, 3, 6, 12, and 24 h post‐infestation (n = 8 biological replicates). In the transgenic plant assay, WT and *TaCRVP*‐OE tomato plants were infested with third‐instar larvae, and samples were harvested at 0, 3, and 6 h post‐infestation (n = 3 biological replicates). All harvested samples were immediately flash‐frozen in liquid nitrogen and ground into a fine powder. For phytohormone extraction, approximately 100 mg of powdered tissue per replicate was homogenized in an ice‐cold extraction solvent (methanol:water:formic acid, 80:19:1, v/v/v) containing stable isotope‐labeled internal standards (D_6_‐SA, D_6_‐JA, and ^1^ ^3^C_6_‐JA‐Ile). Following vortexing, ultrasonication, and centrifugation, the supernatant was evaporated to dryness under a gentle nitrogen stream and reconstituted in the initial mobile phase. The concentrations of JA, JA‐Ile, and SA (SA was quantified only in the insect‐treatment assay) were subsequently determined via LC‐MS/MS at the Testing Center of Fujian Agriculture and Forestry University (Fuzhou, China).

### 4.14 Transcriptomic and Metabolomic Sequencing

Six‐week‐old WT and *TaCRVP*‐OE tomato plants were infested with third‐instar *T. absoluta* larvae for 2 h. Leaves adjacent to the feeding sites, alongside uninfested control, were collected and immediately flash‐frozen in liquid nitrogen (n = 3 biological replicates per genotype and treatment). Frozen samples were shipped on dry ice to Novogene Co., Ltd. (Beijing, China) for transcriptomic sequencing and metabolomic profiling. Following data acquisition, raw sequencing reads (FASTQ files) and metabolite quantification matrices were processed for differential expression and differential abundance analyses, respectively. Downstream bioinformatic analyses, including PCA, hierarchical clustering, Venn diagram construction, and KEGG pathway enrichment, were systematically performed using the Novogene integrated cloud analysis platform.

### 4.15 Y2H and Y3H Assays

Y2H screening was performed using a tomato cDNA library constructed in the pGADT7 prey vector (Ruiyuan Biotech, Nanjing, China). The full‐length coding sequence of *TaCRVP* was cloned into the pGBKT7 bait vector, and the interaction screening was conducted following the manufacturer's instructions for the Y2HGold‐GAL4 Yeast Two‐Hybrid System (Coolaber Technology, Beijing, China; YH2021). Positive prey clones were subsequently isolated to obtain plasmids for sequencing. To investigate specific interactions, full‐length *TaCRVP*, *SlCAT2*, and *SlAdBiL*, alongside the mutated variants *TaCRVP*_R47A_, *TaCRVP*_Q51A_, *TaCRVP*m1–m3, and *SlCAT2*m1–m3, were subcloned into the pGBKT7 bait vector. Corresponding prey vectors were constructed by inserting full‐length *SlCAT2* or the *N. benthamiana* homolog Nb01049 into pGADT7. Bait and prey plasmids were co‐transformed into *S. cerevisiae* strain AH109 and grown on SD medium lacking leucine and tryptophan (SD/−Leu/−Trp) at 28°C for 3 days. To verify positive interactions,, co‐transformants were plated onto high‐stringency SD medium lacking leucine, tryptophan, histidine, and adenine (SD/−Leu/−Trp/−His/−Ade) supplemented with X‐α‐gal, where successful interactions were indicated by the development of blue colonies.

Y3H assay was performed as previously described [47]. The *SlCAT2* and *TaCRVP* were cloned into the pBridge vector, with *TaCRVP* expression strictly controlled by a methionine‐repressible promoter, while the E3 ligase *SlAdBiL* was inserted into pGADT7. These constructs were co‐transformed into AH109 cells. Protein interactions were evaluated on SD medium lacking leucine, tryptophan, and histidine (SD/−Leu/−Trp/−His) in the presence or absence of methionine, enabling the induction of TaCRVP (in the absence of methionine) to directly assess its effect on the SlCAT2–SlAdBiL interaction. Co‐transformations with pBridge‐SlCAT2‐TaCRVP plus empty pGADT7, and pBridge‐SlCAT2 plus AD‐SlAdBiL, served as negative and positive controls, respectively. All primer sequences used for plasmid construction are listed in Table S1.

### 4.16 GST Pull‐Down Assay

To confirm in vitro protein interactions and evaluate competitive binding dynamics, GST pull‐down assays were performed using recombinant proteins expressed in *E. coli* BL21. For pairwise interactions (confirming SlCAT2 interactions with TaCRVP, TaCRVP_Q51A_, SlAdBiL, and itself), GST‐fused SlCAT2 (in pGEX‐4T‐3) was immobilized on glutathione‐Sepharose beads (GE Healthcare, Uppsala, Sweden; 17075601). for 5 h at 4°C. After centrifugation (500 × *g*, 5 min) and washing, the beads were incubated with His‐tagged TaCRVP, TaCRVP_Q51A_, SlAdBiL, or SlCAT2 (in pET‐28b) for an additional 5 h at 4°C. For competition pull‐down assays (evaluating the effect of TaCRVP on SlCAT2 homodimerization and the SlCAT2‐SlAdBiL interaction), immobilized SlCAT2‐GST was first pre‐incubated with MBP‐tagged SlCAT2 or SlAdBiL for 5 h to allow initial complex formation. Following PBS washes, the resulting bead‐protein complexes were incubated with His‐tagged TaCRVP or GFP (control) for a further 5 h. Across all assays, after a final extensive wash with PBS, co‐precipitated proteins were eluted and analyzed via immunoblotting using anti‐His, anti‐GST, and anti‐MBP antibodies (TransGen Biotech, Beijing, China; HT501, HT601, HT701). All primer sequences used for plasmid construction are listed in Table S1.

### 4.17 Native‐PAGE and Western Blot Analysis of SlCAT2 Multimerization

To investigate the in vivo effect of TaCRVP on SlCAT2 multimerization, native proteins were extracted from *N. benthamiana* leaves transiently expressing SlCAT2 (alone or co‐expressed with *TaCRVP* or *TaCRVP*_Q51A_ via *Agrobacterium*), from WT tomato leaves infested with wild‐type, ds*TaCRVP*‐treated, or ds*GFP*‐treated *T. absoluta* larvae (alongside uninfested controls), and from intact leaves of WT and *TaCRVP*‐OE transgenic tomato plants. Total native proteins were extracted using the NativePAGE Sample Prep Kit (Invitrogen, Carlsbad, CA, USA; BN2008). To preserve multimeric protein complexes, soluble extracts were gently mixed with 2× native loading buffer (0.05 m Tris‐HCl, pH 6.8, 10% glycerol, 0.5% bromophenol blue) strictly without boiling. Samples were loaded onto an 8% native polyacrylamide gel at a constant 80 V, followed by transfer to a 0.45 µm polyvinylidene difluoride (PVDF) membrane at 400 mA for 1.5 h. To prevent heat‐induced protein denaturation, both the electrophoresis and transfer processes were strictly conducted on ice. Membranes were subsequently probed with an anti‐SlCAT2 antibody to visualize both the monomeric and multimeric states of SlCAT2. For loading controls, CBB staining was used as a loading control for the *N. benthamiana* samples, while an anti‐GAPDH antibody (TransGen Biotech, HC301) was served as an internal control for the tomato samples.

### 4.18 In vitro Assays of SlCAT2 Multimerization and Enzyme Activity Induced by TaCRVP

To assess the in vitro effect of TaCRVP on SlCAT2 multimerization and enzymatic activity, a fixed amount of purified SlCAT2 (2 µg) of purified TaCRVP or TaCRVP_Q51A_ in 30 µL reaction buffer (50 mM Tris‐HCl, pH 7.5, 150 mM NaCl, 1 mM DTT) at 25°C for 1 h. For multimerization profiling, the incubated mixtures were combined with a non‐denaturing loading buffer (lacking SDS and β‐mercaptoethanol) and separated on an 8% native polyacrylamide gel without boiling, and transferred to a PVDF membrane. The multimeric and monomeric states of SlCAT2 were subsequently visualized via immunoblotting using an anti‐SlCAT2 antibody. To evaluate the functional consequences of this interaction, parallel co‐incubations were prepared identically, and SlCAT2 catalase activity within the reaction mixtures was immediately quantified post‐incubation using a Catalase Assay Kit according to the manufacturer's protocol.

### 4.19 LCI Assay

LCI assays were used to investigate the in vivo protein interactions among TaCRVP, SlCAT2, and SlAdBiL as previously described method [48]. Full‐length *TaCRVP* and *SlCAT2* were subcloned into the pCAMBIA1300‐nLUC vector, while *SlAdBiL* and *SlCAT2* were inserted into the pCAMBIA1300‐cLUC vector. Following transformation into *A. tumefaciens* GV3101, bacterial suspensions harboring the respective constructs (or empty vector controls) were mixed in pairwise combinations and co‐infiltrated into *N. benthamiana* leaves. At 48 h post‐infiltration, the leaves were sprayed with 1 mM D‐luciferin (Yeasen Biotech, 40901ES01), incubated in the dark for 10 min, and subsequently imaged using a cooled CCD imaging system (VILBER, Ile‐de‐France, France). All primer sequences used for plasmid construction are listed in Table S1.

### 4.20 Subcellular Localization and Co‐Localization Assays

To investigate subcellular localization and co‐localization, the full‐length coding sequences of *TaCRVP* and *SlCAT2* were cloned into the pEarleyGate101(fused with CFP) and pEarleyGate102 (fused with YFP) plant expression vectors, respectively. To identify the specific subcellular compartment of TaCRVP, a specific peroxisome marker construct (PTS1‐YFP) and an empty vector expressing free CFP (as a negative control) were additionally utilized. Following transformation into *A. tumefaciens* strain GV3101, bacterial suspensions (OD_600_ = 1.0) harboring the respective plasmids were mixed in equal volumes. For the individual localization assay, *N. benthamiana* leaves were co‐infiltrated with *Agrobacterium* strains containing TaCRVP‐CFP (or free CFP) alongside the PTS1‐YFP peroxisome marker. For the protein co‐localization assay, leaves were co‐infiltrated with TaCRVP‐CFP and SlCAT2‐YFP. At 48 h post‐infiltration, CFP and YFP fluorescence signals were visualized using a Zeiss LSM 980 confocal laser scanning microscope (Carl Zeiss AG, Jena, Germany). All primer sequences used for plasmid construction are listed in Table S1.

### 4.21 BiFC Assay

To investigate the in vivo effect of TaCRVP on SlCAT2 homodimerization, full‐length *SlCAT2* was cloned into both the cYFP and nYFP expression vectors. Following transformation into *A. tumefaciens* strain GV3101, bacterial suspensions harboring the SlCAT2‐cYFP, SlCAT2‐nYFP, and TaCRVP (at a final OD_600_ of 0, 0.1, 0.5, or 1.0), or the mutant TaCRVP_Q51A_ construct (at a final OD_600_ of 1.0), were mixed and co‐infiltrated into *N. benthamiana* leaves. At 48 h post‐infiltration, reconstituted YFP fluorescence signals were visualized using a Zeiss LSM 980 confocal laser scanning microscope. All primer sequences used for plasmid construction are listed in Table S1.

### 4.22 Co‐IP Assay

Co‐IP assays were used to investigate in vivo protein interactions among TaCRVP, SlCAT2, and SlAdBiL. Full‐length *TaCRVP*, *SlCAT2*, *SlAdBiL*, and the point mutant *TaCRVP*_Q51A_ were subcloned into the pGD expression vectors. Following transformation into *A. tumefaciens* strain GV3101, *N. benthamiana* leaves were co‐infiltrated with *A. tumefaciens* suspensions harboring the respective constructs. At 48 h post‐infiltration, leaves were harvested and homogenized in RIPA lysis buffer (Coolaber Technology, SL1020) supplemented with PMSF (Coolaber Technology, SL1079) using a Tissue Homogenizer (Qiagen, Hilden, Germany). Following centrifugation (12,000 × *g*, 15 min, 4°C), 20 µL of BeyoMag Protein A+G beads (Beyotime Biotechnology, P2108) were washed three times with PBS and pre‐incubated with specific antibodies against GFP (TransGen Biotech, HT801), Flag, or Strep (Sangon Biotech, D191106) for 2 h at 4°C. Following three additional washes, the antibody‐conjugated beads were incubated with the cleared supernatant for 4 h at 4°C. After three extensive washes with PBS, immunoprecipitated proteins were eluted via boiling and analyzed by immunoblotting. All primer sequences used for plasmid construction are listed in Table S1.

### 4.23 Whole‐Mount Immunohistochemistry

Third‐instar *T. absoluta* larvae were allowed to feed on tomato leaves for 24 h, after insect‐damaged leaves and mechanically wounded controls were harvested and fixed in formalin‐acetic acid‐alcohol (FAA) for 4 h. Following dehydration through a graded ethanol series and subsequent rehydration, tissues were blocked for 2 h in PBS supplemented with 1% bovine serum albumin (BSA) prior to overnight incubation at 4°C with a custom rabbit polyclonal anti‐TaCRVP antibody (GenScript Biotech, Nanjing, China). After extensive PBS washes, samples were incubated with an HRP‐conjugated secondary antibody (Servicebio, Wuhan, China; GB23303) for 1 h at room temperature. Following final washes, immunolabeling signals were developed using a DAB substrate, and TaCRVP localization was visualized and imaged via a Pannoramic SCAN II digital slide scanner (3DHISTECH, Budapest, Hungary).

### 4.24 Whole‐Mount Immunofluorescence

To visualize the in vivo secretion and localization of TaCRVP during insect feeding, whole‐mount immunofluorescence assays were performed on tomato leaves. Briefly, tomato leaf tissues subjected to *T. absoluta* feeding (insect wounding) or mechanical damage (mechanical wounding) were excised and immediately fixed in 4% (w/v) paraformaldehyde (PFA) in PBS, for 4 h at 4°C under vacuum infiltration. Following fixation, the samples were washed three times with PBS and permeabilized using 0.5% (v/v) Triton X‐100 (Servicebio, G3068) in PBS for 30 min. The tissues were then blocked with 5% (w/v) BSA in PBS for 1 h at room temperature to eliminate non‐specific staining. For immunodetection, the blocked tissues were incubated with a primary antibody against TaCRVP overnight at 4°C. After three extensive washes with PBS, the samples were incubated with a Cy3‐conjugated secondary antibody (Servicebio, GB21303) for 1 h at room temperature in the dark. Nuclei were subsequently counterstained with DAPI (Servicebio, G1012) for 10 min. Finally, the prepared leaf samples were mounted on glass slides and imaged using a Pannoramic SCAN II digital slide scanner (3DHISTECH, Budapest, Hungary).

### 4.25 In vitro Ubiquitination Assay

The in vitro ubiquitination assay was performed as previously described [49]. Briefly, a 30 µL reaction mixture containing 50 ng of E1 activating enzyme, 100 ng E2 conjugating enzyme (Yeasen Biotech, 20433ES25, 20435ES65), 5 µg HA‐tagged ubiquitin (Solarbio Science & Technology, P20020), 500 ng of SlAdBiL‐GST (E3 ligase), and 2 µg SlCAT2‐His substrate in a reaction buffer (50 mM Tris‐HCl, pH 7.5, 10 mM MgCl_2_, 2 mM DTT, 5 mM ATP), was incubated at 30°C for 1 h. The reactions were terminated via the addition of 5× SDS loading buffer, resolved by 15% SDS‐PAGE, and subsequently analyzed by immunoblotting using anti‐HA (TransGen Biotech, HT301), anti‐His, and anti‐GST antibodies. All primer sequences used for plasmid construction are listed in Table S1.

### 4.26 In vivo Ubiquitination and Degradation Assay

To detect SlAdBiL‐mediated ubiquitination of SlCAT2 in planta, full‐length *TaCRVP*‐Myc, *SlCAT2*‐Strep, *Ub*‐Flag, and *SlAdBiL*‐GFP, alongside the mutant *TaCRVP*_Q51A_‐Myc, were subcloned into the pGD expression vectors. Following transformation into *A. tumefaciens* strain GV3101, *N. benthamiana* leaves were co‐infiltrated with *A. tumefaciens* suspensions harboring the appropriate construct combinations. At 24 h post‐infiltration, leaves were treated with 50 µM MG132 (Sigma‐Aldrich, M7449) for 4 h to inhibit proteasomal degradation [12]. Total proteins were extracted using a Co‐IP lysis buffer supplemented with MG132, and ubiquitinated complexes using Flag‐Trap agarose beads. Following extensive washing, SlCAT2 ubiquitination status was analyzed via immunoblotting with anti‐Strep, anti‐Flag, anti‐Myc (TransGen Biotech, HT101), and anti‐GFP antibodies, with ubiquitination smear integrated densities (n = 3 biological replicates) quantified via ImageJ and normalized to corresponding SlCAT2‐Strep input bands.

To investigate endogenous SlCAT2 ubiquitination in vivo, leaves from WT, *TaCRVP*‐OE, TRV2‐empty, and TRV2‐*SlAdBiL* tomato plants were similarly pre‐treated with 50 µM MG132 for 4 h. The leaf tissues were homogenized in IP lysis buffer containing a protease inhibitor cocktail, and cleared lysates (12,000 × *g*, 15 min, 4°C) were either reserved as input controls or incubated overnight at 4°C with an anti‐SlCAT2 antibody (GenScript Biotech, Nanjing, China). Parallel incubation with normal IgG (TransGen Biotech, HS101) served as a negative control. Protein A/G agarose beads were added for a 2‐h incubation at 4°C. Following three extensive washe, immunoprecipitated proteins were eluted via boiling, and endogenous was analyzed using an anti‐Ubiquitin antibody (Abmart, Shanghai, China; M026378S). Reserved inputs were simultaneously immunoblotted with anti‐SlCAT2 and anti‐GAPDH antibodies to monitor the endogenous SlCAT2 abundance and confirm equal loading. All primer sequences used for plasmid construction are listed in Table S1.

### 4.27 Western Blot

Protein samples were separated via SDS‐PAGE (8.5%, 10%, or 15% gels) and transferred onto methanol‐activated PVDF membranes. Following blocking with 5% non‐fat milk for 1.5 h at room temperature, membranes were incubated overnight at 4°C with specific primary antibodies. Following three washes with PBST, membranes were incubated with HRP‐conjugated secondary antibodies (TransGen Biotech, Beijing, China) for 45 min at room temperature. Chemiluminescent signals were developed using a Super ECL Detection Kit (Herui Biotech, HRD0814), visualized via an ECL Plus Detection System (Cytiva, Marlborough, MA, USA), and quantified using ImageJ software (NIH, Bethesda, MD, USA).

### 4.28 VIGS in S. lycopersicum

To investigate the role of *SlAdBiL* in tomato immunity via VIGS, a specific fragment of the *SlAdBiL* coding sequence was cloned into the pTRV2 vector to generate the TRV2‐*SlAdBiL* construct. Following transformation into *A. tumefaciens* strain GV3101, *A. tumefaciens*. suspensions harboring pTRV1 were mixed at a 1:1 volume ratio with those carrying either TRV2‐*SlAdBiL* or the empty pTRV2 vector (control) and infiltrated into tomato cotyledons using a needleless syringe. Infiltrated seedlings were maintained in a controlled environment chamber (25°C, 16‐h light/8‐h dark photoperiod). Two weeks post‐infiltration, silencing efficiency was confirmed via RT‐qPCR, and successfully knocked‐down plants (alongside TRV2‐empty controls) were subsequently utilized to evaluate tomato resistance against *T. absoluta*. All primer sequences used for plasmid construction are listed in Table S1.

### 4.29 Structural Modeling and Molecular Docking

The 3D structures of TaCRVP and SlCAT2 were predicted using AlphaFold 3 (Google DeepMind, London, UK). Protein‐protein docking was subsequently performed via the HDOCK web server, with the top‐scoring complex conformation selected for downstream evaluation. Interfacial molecular interactions within the predicted complex were assessed using the PDBePISA web server (https://www.ebi.ac.uk/pdbe/pisa/), while all structural visualizations and binding site analyses were executed using PyMOL v3.1 (Schrödinger, LLC, New York, NY, USA).

### 4.30 Phylogenetic Analysis

To evaluate evolutionary conservation and establish a basis for the potential interaction between TaCRVP and *N. benthamiana* catalases, homologous amino acid sequences of *N. benthamiana* catalases and SlCAT2 were retrieved from the NCBI GenBank database, aligned via ClustalW, and subjected to maximum likelihood (ML) phylogenetic tree construction in MEGA7 with 1000 bootstrap replicates.

### 4.31 Statistical Analysis

All statistical analyses were conducted using GraphPad Prism 9.0 (GraphPad Software, San Diego, CA, USA) and SPSS Statistics 21.0 (IBM Corp., Armonk, NY, USA), following signal quantification via ImageJ software. Differences between two independent groups were evaluated using two‐tailed Student's *t*‐tests, while comparisons among three or more groups were analyzed via one‐way or two‐way analysis of variance (ANOVA) followed by Tukey's HSD *post hoc* test. Data are expressed as means ± standard deviation (SD) or standard error (SE), with statistical significance defined as *p* < 0.05. Specific statistical methods, error bar definitions, and sample sizes for individual experiments are detailed in the corresponding figure legends.

## Author Contributions

Y.H., L.P., J.Y., Y.Z. and Z.W. designed the research; J.Y., Y.Z. and Z.W. performed the experiments and analyzed the data; H.T., H.L., Z.C., S.L. and J.S. analyzed the data; J.Y., Y.Z. and Z.W. wrote the paper; Y.H. and L.P. contributed to the critically revising of the manuscript.

## Funding

This work was supported by the National Natural Science Foundation of China (Grants U22A20489 and 32361143791).

## Conflicts of Interest

The authors declare that they have no competing interests.

## Supporting information




**Supporting File 1**: advs77331‐sup‐0001‐SuppMat.xlsx.

**Supporting File 2**: advs77331‐sup‐0002‐SuppMat.docx.

**Supporting File 3**: advs77331‐sup‐0003‐TableS1.xlsx.

**Supporting File 4**: advs77331‐sup‐0004‐TableS2.xlsx.

**Supporting File 4**: advs77331‐sup‐0005‐TableS3.xlsx.

## Data Availability

The datasets generated and analyzed during the current study are available in public repositories. The transcriptomic sequencing data have been deposited in the NCBI Gene Expression Omnibus (GEO) database under the accession number GSE332550. The mass spectrometry proteomics data have been deposited to the ProteomeXchange Consortium via the PRIDE partner repository with the dataset identifier PXD078333. The metabolomics data have been deposited in the MetaboLights repository under the accession number MTBLS14509. All other data supporting the findings of this study are available within the paper and its Supplementary Information files.
