# Supplementary material for: An Effector TaCRVP From Tuta Absoluta Oral Secretion Impairs H2O2‐Mediated Plant Defense by Targeting Tomato SlCAT2
Source: Adv Sci (Weinh). 2026 Aug 25;13(63):e77331. doi: 10.1002/advs.77331 (PMC13505802; doi:10.1002/advs.77331)
Supplement: Supplementary file 1 [file ADVS-13-e77331-s002.docx]

**Supporting Information**

**An effector TaCRVP from *Tuta absoluta* oral secretion impairs H₂O₂-mediated plant defense by targeting tomato SICAT2**

*Jianquan Yan^#^, Youdan Zhang^#^, Zhujun Wang^#^, Huatao Tang, Huiping Liu, Zi Chen, Shuai Liu, Jiayi Shi, Lu Peng*, and Youming Hou**

State Key Laboratory of Agricultural and Forestry Biosecurity, Fujian Agriculture and Forestry University, Fuzhou, Fujian, China

**Figure S1. Proteomic identification of** **candidate venom proteins from the oral secretions (OSs) of *T. absoluta*.** (A) Protein profile of *T. absoluta* OSs visualized by SDS-PAGE and Coomassie Brilliant Blue (CBB) staining. OSs were collected and pooled from third- and fourth-instar larvae. The complex banding pattern indicates the presence of a diverse protein repertoire in the OSs. M, molecular weight marker (kDa). The original blots can be found in Supplementary Figure S7. (B) Identification of the two candidate venom proteins via LC-MS/MS analysis of the *T. absoluta* OSs proteome. The identified peptides and corresponding protein parameters are presented. Sequence coverage (%) represents the proportion of the target protein successfully sequence matched by the identified peptides relative to its full-length sequence in the *T. absoluta* reference database.

**Figure S2. Transient expression of the salivary effector TaCRVP suppresses early defense responses and promotes *T. absoluta* feeding in *N. benthamiana*.** (A) Immunoblot analysis confirming the expression and integrity of TaCRVP-GFP and GFP control in *N. benthamiana* leaves. Total proteins were extracted at 48 h post-agroinfiltration and detected using an anti-GFP antibody. The original blots can be found in Supplementary Figure S8. (B) Bioassays of *T. absoluta* larvae. (B-i) Leaf consumption area and body weight of *T. absoluta* larvae following 48 h of feeding on *N. benthamiana* leaves transiently expressing TaCRVP or the GFP control (n = 17–20). (B-ii) Representative images of the corresponding larvae after the 48-h feeding. Scale bars = 1 cm. (C) TaCRVP suppresses the flg22-triggered oxidative burst in *N. benthamiana*. (C-i) *N. benthamiana* leaves were co-infiltrated with GFP or TaCRVP *Agrobacterium* suspensions mixed with flg22. At 48 h post-infiltration, leaves were stained with DAB to visualize cumulative H₂O₂ accumulation. Co-infiltration with flg22 treatment serving as a positive control. Scale bars = 1 cm. (C-ii) Quantification of total H₂O₂ accumulation in the co-infiltrated leaves using a hydrogen peroxide assay kit. (D) Dynamic monitoring of flg22-triggered ROS burst. Continuous ROS production was measured using a luminol-based chemiluminescence assay in *N. benthamiana* leaf disks transiently expressing TaCRVP or a GFP, following treatment with flg22 or H₂O. Data represent the mean ± SE (n = 12; RLU, relative light units). Data are presented as mean ± SD unless otherwise noted. Statistical significance was determined using two-tailed Student's *t*-tests (B) (ns, not significant; *, *P* < 0.05; **, *P* < 0.01; ***, *P* < 0.001; ****, *P* < 0.0001). Multiple comparisons were analyzed using one-way ANOVA followed by Tukey's HSD test (C). Different lowercase letters indicate statistically significant differences based on ANOVA (*P* < 0.05).

**Figure S3. Signal peptide and transmembrane domains of TaCRVP predicted by SignalP-6.0 and TMHMM 2.0.** (A) Prediction of the TaCRVP secretory signal peptide using the SignalP-6.0 server. SP (Sec/SPI) denotes the probability of a standard secretory signal peptide (translocated by the Sec translocon and cleaved by Signal Peptidase I), CS indicates the predicted cleavage site, and OTHER represents the probability of the sequence lacking a signal peptide. The predicted signal peptide likelihood is 0.9996, with the precise cleavage site identified between amino acid residues 19 and 20 (SQA-SV) with a probability of 0.9687. (B) Transmembrane topology prediction using the TMHMM 2.0 server. The graph displays the posterior probability of each amino acid residue being located inside the cell (blue line), outside the cell (yellow line), or embedded within a transmembrane helix (purple line). The probability for the transmembrane state remains near zero throughout the full-length sequence, while the outside state predominates, confirming the complete absence of transmembrane domains and structurally supporting its identity as a fully secreted protein.

**Figure S4. LC-MS/MS proteomic analysis confirms the presence of the TaCRVP effector in the OSs of *T. absoluta*.** (A) The complete amino acid sequence of TaCRVP protein. The specific sequence regions corresponding to the unique peptides successfully captured and identified via LC-MS/MS analysis from the *T. absoluta* OS proteome are highlighted in red. (B) Representative tandem mass spectra (MS/MS) of the two unique peptides used for the unambiguous identification of TaCRVP in the OSs. The comprehensively annotated b-ion (amino-terminal) and y-ion (carboxy-terminal) fragmentation series definitively confirm the precise amino acid sequences of unique peptide 1 (TASNLFTK) and unique peptide 2 (TASNLFTKPK).

**Figure S5. Specificity validation of the TaCRVP polyclonal antibody.** (A) Immunoblot analysis confirming the high specificity of the generated anti-TaCRVP polyclonal antibody. Lane 1: purified recombinant TaCRVP protein expressed in *E. coli* strain BL21; Lane 2: total cell lysate from the empty vector control; Lane 3: total endogenous protein extracted from *T. absoluta* larvae. The detection of distinct, single bands corresponding to the expected molecular weights in Lanes 1 and 3, coupled with the absolute absence of non-specific signals in Lane 2, demonstrates the high specificity of the antibody for both recombinant and endogenous TaCRVP. (B) Immunoblot detection of endogenous TaCRVP protein levels in *T. absoluta* at 6, 12, 24, and 48 h post-treatment with ds*GFP* or ds*TaCRVP*. β-Tubulin serves as the loading control. The original images for all immunoblots shown in this figure are provided in Supplementary Figure S9.

**Figure S6. Whole-mount immunohistochemical detection of TaCRVP at *T. absoluta* feeding sites on tomato leaves.** Whole-mount immunohistochemical localization of the TaCRVP effector at feeding sites on tomato leaves. Tomato leaves were infested for 24 h with third-instar *T. absoluta* larvae treated with ds*GFP* (control) or ds*TaCRVP*. Mechanical wounding sites on leaves without larval infestation served as a negative control to rule out non-specific background signals induced by tissue damage. The secreted TaCRVP was specifically detected in planta using a custom rabbit polyclonal anti-TaCRVP primary antibody, followed by an HRP-conjugated secondary antibody and DAB chromogenic substrate development. Boxed areas in the main panels indicate the regions shown at higher magnification in the adjacent panels to better highlight the spatial deposition of the effector. Scale bars = 10 μm.

**Figure S7. Validation of systemic dsRNA uptake and temporal dynamics of RNAi-mediated gene silencing in *T. absoluta*.** (A) Fluorescence visualization of the whole larva, as well as the dissected salivary glands and gut, at 12 h following a 10-min soaking treatment with fluorescent dsRNA. The widespread accumulation of fluorescent signals confirms successful systemic uptake and delivery of the dsRNA into target tissues. (B) Temporal dynamics of TaCRVP RNAi efficiency. The relative transcript levels of *TaCRVP* in larvae were quantified by RT-qPCR at 6, 12, 24, 48, 72, 96, and 120 h following the 10-min soaking treatment with ds*TaCRVP* or ds*GFP*. The expression levels were normalized to the internal reference gene *EF1α*. Data are presented as mean ± SD (n = 3). Statistical significance was determined using two-tailed Student's *t*-tests (ns, not significant; *, *P* < 0.05; **, *P* < 0.01; ***, *P* < 0.001; ****, *P* < 0.0001).

**Figure S8. Effect of *TaCRVP* silencing on the body weight of *T. absoluta* larvae** **reared on an artificial diet.** Body weight of *T. absoluta* larvae was recorded following 48 h of feeding exclusively on an artificial diet. Prior to the feeding assay, the larvae were subjected to RNAi treatment with ds*TaCRVP* or ds*GFP* via a 10-min soaking treatment. Data are presented as mean ± SD (n = 16–17). Statistical significance was determined by two-tailed Student's *t*-tests (ns, not significant; *, *P* < 0.05; **, *P* < 0.01; ***, *P* < 0.001; ****, *P* < 0.0001).

**Figure S9. Generation and phenotypic characterization of transgenic *S. lycopersicum* lines** **overexpressing *TaCRVP*.** (A) Immunoblot analysis confirming the successful expression of the TaCRVP protein in the transgenic tomato lines (*TaCRVP*-OE). Total proteins were extracted from the leaves of wild-type (WT) and *TaCRVP*-OE, and the Flag-tagged TaCRVP was detected using an anti-Flag antibody. CBB staining serves as the loading control. The original blots can be found in Supplementary Figure S10. (B) Phenotypic comparison of WT and *TaCRVP*-OE tomato plants. The transgenic lines exhibit no obvious differences in growth and development compared to the WT. Scale bars = 5 cm. (C) Comparison of plant height between WT and *TaCRVP*-OE transgenic tomato plants. Data are presented as mean ± SD (n = 13). Statistical significance was determined using two-tailed Student's *t*-tests (ns, not significant; *, *P* < 0.05; **, *P* < 0.01; ***, *P* < 0.001; ****, *P* < 0.0001).

**Figure S10. RNA-seq analysis reveals that the salivary effector TaCRVP induces global transcriptional reprogramming in *S. lycopersicum*.** To assess the impact of TaCRVP on plant global transcription, leaf samples from four experimental groups were subjected to RNA-seq analysis (n = 3): untreated WT tomato plants (WT), untreated *TaCRVP*-OE tomato plants (*TaCRVP*_OE), WT plants following 2 h of feeding by *T. absoluta* larvae (WT_Inf), and *TaCRVP*-OE plants following 2 h of *T. absoluta* feeding (*TaCRVP*_OE_Inf). (A) Hierarchical clustering heatmap illustrating the expression profiles of all differentially expressed genes across the four experimental groups. Gene expression levels are presented as the mean log_10_ (FPKM+1) values. The color scale indicates relative expression levels, with red and blue representing higher and lower transcript abundances, respectively. (B) Principal component analysis (PCA) of the global transcriptome data demonstrating the tight clustering of biological replicates and distinct separation among different genotypes and treatments. PC1 accounts for 35.33% of the variance, and PC2 accounts for 25.04%.

**Figure S11. TaCRVP attenuates the induction of defense-related genes within the Inf_UP cluster during *T. absoluta* feeding.** (A) RNA-seq expression profiles of defense-related genes from the Inf_UP group (a cluster of genes typically upregulated upon herbivore infestation). Transcript abundances in the leaves of WT and *TaCRVP*-OE tomato plants are presented as FPKM (fragments per kilobase of exon model per million mapped fragments). Untreated plants from both genotypes served as the baseline control (CK). (B) RT-qPCR validation of selected defense-related differentially expressed genes (DEGs) from the Inf_UP group. Leaf samples were collected from CK and herbivore-infested plants at 2 h post-infestation by *T. absoluta* larvae. Relative expression levels were normalized to the internal reference gene *SlActin*. Data are presented as mean ± SD (n = 3). Statistical significance was determined using two-way ANOVA followed by Tukey's HSD test (ns, not significant; *, *P* < 0.05; **, *P* < 0.01; ***, *P* < 0.001; ****, *P* < 0.0001).

**Figure S12.** **Widely targeted metabolomics analysis reveals metabolic reprogramming mediated by TaCRVP in *S. lycopersicum* leaves.** (A) PCA of the global metabolomics data, demonstrating the tight clustering of biological replicates and distinct metabolic separation among different genotypes and treatments. PC1 accounts for 23.51% of the total variance, and PC2 accounts for 20.23%. Group definitions are as follows: untreated WT tomato plants (WT), untreated *TaCRVP*_OE plants (*TaCRVP*_OE), WT plants following 2 h of feeding by *T. absoluta* larvae (WT_Inf), and *TaCRVP*-OE plants following 2 h of *T. absoluta* feeding (*TaCRVP*_OE_Inf). (B) Venn diagram illustrating the overlap of TaCRVP-affected differentially accumulated metabolites (DAMs) across the different comparative groups. (C) Classification and relative abundance analysis of the DAMs assigned to the Inf_UP group. This group represents a specific cluster of metabolites typically induced upon herbivore infestation. (D) KEGG pathway enrichment analysis of the specific metabolites within the Inf_UP group described in (C). The size of the circles represents the number of enriched metabolites, and the color gradient Indicates the statistical significance (*P*-value).

**Figure S13.** **Identification of putative tomato interacting proteins of the TaCRVP effector through Y2H screening.** A *S. lycopersicum* cDNA library was screened using the TaCRVP as the bait. The table summarizes the putative interacting candidates identified from the initial screening. "Gene ID / Accession Number" and "Protein Annotation" correspond to the *S. lycopersicum* reference genome database. "Number of Clones" indicates the number of independent positive yeast colonies recovered and sequenced for each respective candidate gene on the selective medium SD/-Trp/-Leu/-His/-Ade supplemented with X-α-Gal.

**Figure S14.** **TaCRVP mutants** **differ in their ability to suppresses the flg22-triggered oxidative burst in *N. benthamiana*.** (A) The TaCRVPm2 mutant suppresses the flg22-triggered oxidative burst in *N. benthamiana*. (A-i) *N. benthamiana* leaves were co-infiltrated with GFP, TaCRVP, or TaCRVP mutants *Agrobacterium* suspensions mixed with flg22. At 48 h post-infiltration, leaves were stained with DAB to visualize cumulative H₂O₂ accumulation, with flg22 treatment serving as a positive control. Scale bars = 1 cm. (A-ii) Quantification of total H₂O₂ accumulation in the co-infiltrated leaves using a hydrogen peroxide assay kit (n = 3). (B) Dynamic monitoring of flg22-triggered ROS burst. Continuous ROS production was measured using a luminol-based chemiluminescence assay in *N. benthamiana* leaf disks transiently expressing TaCRVP or TaCRVP mutants, following treatment with flg22 or H₂O. Data represent the mean ± SE (n = 12; RLU, relative light units). (C) The TaCRVP_Q51A_ mutant failed to suppress the flg22-triggered oxidative burst in *N. benthamiana* leaves. (C-i) *N. benthamiana* leaves were co-infiltrated with GFP, TaCRVP, or TaCRVPQ51A *Agrobacterium* suspensions mixed with flg22. At 48 h post-infiltration, leaves were stained with DAB to visualize cumulative H₂O₂ accumulation, with flg22 treatment serving as a positive control. Scale bars = 1 cm. (C-ii) Quantification of total H₂O₂ accumulation in the co-infiltrated leaves using a hydrogen peroxide assay kit (n = 3). (D) Dynamic monitoring of flg22-triggered ROS burst. Continuous ROS production was measured using a luminol-based chemiluminescence assay in *N. benthamiana* leaf disks transiently expressing TaCRVP or TaCRVP_Q51A_, following treatment with flg22 or H₂O. Data represent the mean ± SE (n = 12; RLU, relative light units). Data are presented as mean ± SD unless otherwise noted. Statistical significance was determined using one-way ANOVA followed by Tukey's HSD test. Different lowercase letters indicate statistically significant differences (*P* < 0.05).

**Figure S15. Y2H assays confirm the interactions between TaCRVP and members of the tomato catalase family (SlCAT1,** **SlCAT2, and SlCAT3).** Yeast cells co-transformed with the indicated bait constructs (BD-TaCRVP) and prey constructs (AD-SlCATs) were spotted onto selective media. Cell growth on the double dropout medium (DDO, SD/–Trp/–Leu) confirms successful co-transformation, whereas growth on the quadruple dropout medium (QDO, SD/–Trp/–Leu/–His/–Ade) supplemented with X-α-Gal indicates physical protein-protein interactions. Notably, yeast cells co-expressing BD-TaCRVP and AD-SlCAT2 exhibited more robust growth and a deeper blue coloration compared to those expressing AD-SlCAT1 or AD-SlCAT3, suggesting a stronger interaction. Serially diluted yeast cultures (1, 10⁻¹, and 10⁻²) are shown for each combination. Co-transformants carrying pGBKT7-53/pGADT7-T and pGBKT7-Lam/pGADT7-T served as the positive (+) and negative (–) controls, respectively.

**Figure S16. TaCRVP interacts with *N. benthamiana* catalase** **Nb_01049.** (A) Amino acid sequence alignment of SlCAT2 and *N. benthamiana* catalases. Sequences alignment was performed using Clustal Omega. (B) Phylogenetic analysis of SlCAT2 and *N. benthamiana* catalases. The phylogenetic tree was constructed using the neighbor-joining (NJ) method. The SlCAT2 protein is indicated by a red circle. Numbers at the nodes represent bootstrap confidence values based on 1000 replicates. Branch lengths indicate evolutionary distances calculated using the *p*-distance method. (C) Y2H assay confirms the direct interaction between TaCRVP and Nb_01049. Yeast cells co-transformed with the bait construct (BD-TaCRVP) and prey construct (AD-Nb_01049) were spotted onto selective media. Cell growth on the double dropout medium (DDO, SD/–Trp/–Leu) confirms successful co-transformation. The growth and blue coloration of yeast colonies on the quadruple dropout medium (QDO, SD/–Trp/–Leu/–His/–Ade) supplemented with X-α-Gal indicates a physical protein-protein interaction. Serially diluted yeast cultures (1, 10⁻¹, and 10⁻²) are shown. Co-transformants carrying corresponding empty AD or BD vectors served as auto-activation controls. The combinations of pGBKT7-53/pGADT7-T and pGBKT7-Lam/pGADT7-T served as the positive (+) and negative (–) controls, respectively.

**Figure S17.** **Generation and phenotypic characterization of *SlCAT2*-OE and *SlCAT2*-KN transgenic *S. lycopersicum* lines.** (A) RT-qPCR analysis of *SlCAT2* expression in WT and *SlCAT2*-OE lines (n = 3). Expression levels were normalized to the internal reference gene *SlActin*. (B) Schematic diagram of the CRISPR/Cas9 target site within the *SlCAT2* exon and sequence alignment of the WT and knockout lines (KN-1 to KN-5). Dashes indicate nucleotide deletions, which lead to a frameshift mutation and premature translational termination. (C) Phenotypic comparison of WT, *SlCAT2*-OE, and *SlCAT2*-KN tomato plants. Scale bars = 5 cm. (D) Plant height comparison of WT, *SlCAT2*-OE and *SlCAT2*-KN tomato plants (n = 15). Data are presented as mean ± SD. For (D), statistical significance was determined using one-way ANOVA followed by Tukey's HSD test. Different lowercase letters indicate statistically significant differences (*P* < 0.05).

**Figure S18. Differential effects of *TaCRVP* silencing on *T. absoluta* performance across varying *SlCAT2* genetic backgrounds.** (A) Fold change in larval leaf consumption area. (B) Fold change in larval body weight. The fold changes were calculated as the ratio of the respective performance parameters of ds*TaCRVP*-treated larvae relative to those of the ds*GFP*-treated controls when feeding onWT, *SlCAT2*-OE, and *SlCAT2*-KN tomato plants. Data are presented as mean ± SD (n = 16–19). Statistical significance was determined using one-way ANOVA followed by Tukey’s HSD test. Different lowercase letters indicate statistically significant differences (*P* < 0.05).

**Figure S19.** **SlCAT2 interacts with SlAdBiL in planta.** (A) Co-immunoprecipitation (Co-IP) assay confirming the interaction between SlCAT2 and SlAdBiL. *N. benthamiana* leaves were agroinfiltrated to transiently co-express the indicated fusion proteins SlAdBiL-GFP and SlCAT2-Strep. Total protein extracts (Input) were subjected to immunoprecipitation (IP) using anti-GFP agarose beads. Both the Input and IP fractions were analyzed by immunoblotting using anti-GFP and anti-Strep antibodies. The original blots can be found in Supplementary Figure S11. (B) Luciferase complementation imaging (LCI) assay validating the interaction between SlCAT2 and SlAdBiL in vivo. *N. benthamiana* leaves were co-infiltrated with *Agrobacterium* suspensions harboring the indicated nLuc and cLuc fusion constructs SlCAT2-nLuc and SlAdBiL-cLuc. Luminescence signals were captured at 48 h post-infiltration after the application of D-luciferin. Co-expression of the target proteins with corresponding empty vectors served as negative controls.

**Figure S20. Virus-induced gene silencing (VIGS)-mediated silencing efficiency of *SlAdBiL* in tomato plants.** The relative transcript level of *SlAdBiL* in the leaves of control (TRV2-empty) and *SlAdBiL*-silenced (TRV2-*SlAdBiL*) tomato plants were quantified by RT-qPCR at 2 weeks post-agroinfiltration. Expression levels were normalized to the internal reference gene *SlActin*. Data are presented as mean ± SD (n = 3). Statistical significance was determined using two-tailed Student's *t*-tests (ns, not significant; *, *P* < 0.05; **, *P* < 0.01; ***, *P* < 0.001; ****, *P* < 0.0001).

**Figure S21. Silencing of *SlAdBiL* alters ROS homeostasis and the flg22-triggered oxidative burst in tomato plants.** (A) H₂O₂ accumulation and CAT activity in the leaves of control (TRV2-empty) and *SlAdBiL*-silenced (TRV2-*SlAdBiL*) tomato plants following 3 h of feeding by *T. absoluta* larvae. (n = 3). (B) Dynamic monitoring of flg22-triggered ROS burst. Continuous ROS production was measured using a luminol-based chemiluminescence assay in TRV2-empty and TRV2-*SlAdBiL* tomato leaf disks, following treatment with flg22 or H₂O. Data represent the mean ± SE (n = 12; RLU, relative light units). Statistical significance was determined by two-tailed Student's *t*-tests (ns, not significant; *, *P* < 0.05; **, *P* < 0.01; ***, *P* < 0.001; ****, *P* < 0.0001).

**Figure S22.** **In vitro catalase activity of the recombinant SlCAT2 protein.** In vitro enzymatic activity of the purified SlCAT2-His fusion protein. The recombinant SlCAT2-His and the empty His tag were expressed in *E. coli* strain BL21. CAT activity was measured by monitoring the decomposition of H₂O₂ using a catalase assay kit. Data are presented as mean ± SD (n = 3). Statistical significance was determined using two-tailed Student's *t*-tests (ns, not significant; *, *P* < 0.05; **, *P* < 0.01; ***, *P* < 0.001; ****, *P* < 0.0001).

**Figure S23.** **TaCRVP enhances SlCAT2 enzymatic activity to promote *T. absoluta* performance in *N. benthamiana*.** (A) CAT activity in *N benthamiana* leaves transiently co-expressing SlCAT2 with TaCRVP or the point mutant TaCRVP_Q51A_. Assays were performed at 48 h post-infiltration (n = 3). (B) Bioassays of *T. absoluta* larvae. (B-i) Leaf consumption area and body weight of *T. absoluta* larvae following 48 h of feeding on *N. benthamiana* leaves described in (A) (n = 19–20). (B-ii) Representative images of the corresponding larvae after the 48-h feeding. Scale bars = 1 cm. Data are presented as mean ± SD. Statistical significance was determined using one-way ANOVA followed by Tukey's HSD test. Different lowercase letters indicate statistically significant differences (*P* < 0.05).

**Figure S24.** **SlCAT2 self-interacts in vitro and in planta.** (A) Y2H assay confirms the self-interaction of SlCAT2. Yeast cells co-transformed with the bait construct (BD-SlCAT2) and prey construct (AD-SlCAT2) were spotted onto selective media. Cell growth on the double dropout medium (DDO, SD/–Trp/–Leu) confirms successful co-transformation. The growth and blue coloration of yeast colonies on the quadruple dropout medium (QDO, SD/–Trp/–Leu/–His/–Ade) supplemented with X-α-Gal indicates a physical protein-protein interaction. Serially diluted yeast cultures (1, 10⁻¹, and 10⁻²) are shown. Co-transformants carrying corresponding empty AD or BD vectors served as auto-activation controls. The combinations of pGBKT7-53/pGADT7-T and pGBKT7-Lam/pGADT7-T served as the positive (+) and negative (–) controls, respectively. (B) GST pull-down assay confirms the direct in vitro self-interaction of SlCAT2. Recombinant SlCAT2-GST and SlCAT2-His proteins were expressed in and purified from *E. coli* strain BL21. The purified proteins were incubated together, and the pull-down fractions along with the Input samples were analyzed via immunoblotting using anti-GST and anti-His antibodies. (C) Co-IP assay confirms the in planta self-interaction of SlCAT2. *N. benthamiana* leaves were agroinfiltrated to transiently co-express SlCAT2-GFP and SlCAT2-Strep. Input were subjected to IP using anti-GFP agarose beads. Both the Input and IP fractions were analyzed by immunoblotting using anti-GFP and anti-Strep antibodies. (D) LCI assay validates the self-interaction of SlCAT2 in vivo. *N. benthamiana* leaves were co-infiltrated with *Agrobacterium* suspensions harboring the SlCAT2-nLuc and SlCAT2-cLuc fusion constructs. Luminescence signals were captured at 48 h post-infiltration after the application of D-luciferin. Co-expression of the target proteins with corresponding empty vectors served as negative controls. The original images for all immunoblots shown in this figure are provided in Supplementary Figure S12.

**Figure S25.** **The catalase domain mediates SlCAT2 self-interaction.** (A) Schematic representation of full-length SlCAT2 protein and its truncated mutants. (B) Y2H assays evaluating the interactions between full-length SlCAT2 and its truncated mutants. Yeast cells co-transformed with the indicated bait and prey constructs were spotted onto selective media. Cell growth on the double dropout medium (DDO, SD/–Trp/–Leu) confirms successful co-transformation. The growth and blue coloration of yeast colonies on the quadruple dropout medium (QDO, SD/–Trp/–Leu/–His/–Ade) supplemented with X-α-Gal indicates physical protein-protein interactions. Serially diluted yeast cultures (1, 10⁻¹, and 10⁻²) are shown. Co-transformants carrying corresponding empty AD or BD vectors served as auto-activation controls. The combinations of pGBKT7-53/pGADT7-T and pGBKT7-Lam/pGADT7-T served as the positive (+) and negative (–) controls, respectively.

**Figure S26. TaCRVP promotes the self-interaction of SlCAT2 in planta.** Co-IP assay demonstrating the enhancement of SlCAT2 self-interaction by TaCRVP. *Nicotiana benthamiana* leaves were agroinfiltrated to transiently co-express SlCAT2-Flag and SlCAT2-Strep, in the presence of either TaCRVP-GFP or the free GFP control. Input were subjected to IP using anti-Flag agarose beads. Both the Input and IP fractions were analyzed by immunoblotting using anti-Flag, anti-Strep, and anti-GFP antibodies to detect the respective proteins. The original blots can be found in Supplementary Figure S13.

**Figure S27.** **Transcript levels of *SlCAT2* in WT and *TaCRVP*-OE tomato plants.** RT-qPCR analysis of relative *SlCAT2* expression in the leaves of WT and *TaCRVP*-OE tomato lines. Expression levels were normalized to the internal reference gene *SlActin*.

Data are presented as mean ± SD (n = 3). Statistical significance was determined using two-tailed Student's *t*-tests (ns, not significant; *, *P* < 0.05; **, *P* < 0.01; ***, *P* < 0.001; ****, *P* < 0.0001).

**Figure S28.** **Taxonomic composition of the customized Lepidoptera protein reference database used for proteomic analysis.** The table details the taxonomic classification (Order, Family, and Genus) and the corresponding number of protein sequences for each taxon included in the database. To maximize the identification rate of LC-MS/MS spectra, this comprehensive reference database was constructed by integrating available sequences from *Tuta* and multiple related lepidopteran genera.
